# PP2A inhibition causes synthetic lethality in *BRCA2*-mutated prostate cancer models via spindle assembly checkpoint reactivation

**DOI:** 10.1172/JCI172137

**Published:** 2024-01-02

**Authors:** Jian Wang, Yuke Chen, Shiwei Li, Wanchang Liu, Xiao Albert Zhou, Yefei Luo, Zhanzhan Xu, Yundong Xiong, Kaiqi Cheng, Mingjian Ruan, Wei Yu, Xiaoman Li, Weibin Wang, Jiadong Wang

**Affiliations:** 1 Department of Radiation Medicine, School of Basic Medical Sciences, Peking University International Cancer Institute, Beijing Key Laboratory of Tumor Systems Biology, Peking University Health Science Center, Beijing, China.; 2 Department of Urology, Peking University First Hospital, Beijing, China.

**Keywords:** Cell Biology, Oncology, DNA repair, Prostate cancer, Tumor suppressors

## Abstract

Mutations in the *BRCA2* tumor suppressor gene have been associated with an increased risk of developing prostate cancer. One of the paradoxes concerning BRCA2 is the fact that its inactivation affects genetic stability and is deleterious for cellular and organismal survival, while *BRCA2*-mutated cancer cells adapt to this detriment and malignantly proliferate. Therapeutic strategies for tumors arising from *BRCA2* mutations may be discovered by understanding these adaptive mechanisms. In this study, we conducted forward genetic synthetic viability screenings in *Caenorhabditis* *elegans brc-2* (*Cebrc-2*) mutants and found that *Ceubxn-2* inactivation rescued the viability of *Cebrc-2* mutants. Moreover, loss of NSFL1C, the mammalian ortholog of CeUBXN-2, suppressed the spindle assembly checkpoint (SAC) activation and promoted the survival of BRCA2-deficient cells. Mechanistically, NSFL1C recruited USP9X to inhibit the polyubiquitination of AURKB and reduce the removal of AURKB from the centromeres by VCP, which is essential for SAC activation. SAC inactivation is common in BRCA2-deficient prostate cancer patients, but PP2A inhibitors could reactivate the SAC and achieve BRCA2-deficient prostate tumor synthetic lethality. Our research reveals the survival adaptation mechanism of BRCA2-deficient prostate tumor cells and provides different angles for exploring synthetic lethal inhibitors in addition to targeting DNA damage repair pathways.

## Abstract 2

Survival screenings of C. elegans identifies pathways and genes responsible for BRCA2-induced lethality in prostate cancer models.

## Graphical abstract

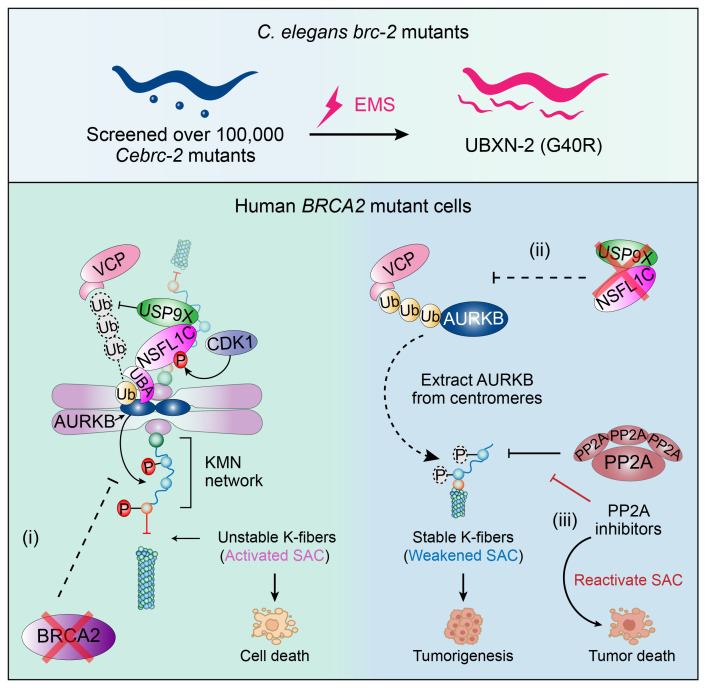

## Introduction

Germline mutations of *BRCA2* predispose humans to prostate, breast, ovarian, and pancreatic cancers (1, 2). As a tumor suppressor protein, BRCA2 is essential for stabilizing stalled replication forks (2, 3) and promoting the repair of replication-related DNA damage by homologous recombination (HR) (2), and for the role it plays in chromosome segregation during mitosis (1, 4, 5). As a result, BRCA2-deficient tumor cells need to partially restore these functions or possess alternative repair pathways to survive, owing to highly unstable genomes and chromosomes.

The role of BRCA2 in cancer susceptibility is poorly understood. Mice homozygous for *Brca2* exon 10 and 11 truncating mutations exhibit embryonic lethality (6–8), whereas a minority of homozygous mice harboring a *Brca2* truncation at the 3′ end of exon 11 survive to adulthood and develop thymic lymphomas (9, 10). One of the paradoxes concerning BRCA2 is that it is not only necessary during biological development, where its absence leads to embryonic lethality, but also is essential for cell viability (6–11). How these BRCA2-deficient tumor cells survive despite accumulating DNA damage and abnormal mitosis is not fully understood, and this lethality must be overcome during tumorigenesis. In fact, concomitant deletion of *p53* in mice delays early embryonic lethality in *Brca2^−/−^* embryos, but, apart from *p53* mutations (8), little is known about other mechanisms that enable *Brca2^−/−^* embryos to develop. Recent work on BRCA2 shows that protecting replication forks from degradation can rescue the viability of *Brca2*-knockout mouse embryonic stem cells (12, 13) and the inactivation of *p53* or *Bub1* can reverse the growth arrest in *Brca2*-deficient mouse embryonic fibroblasts (14). These observations indicate that BRCA2-deficient cells adapt to severe genomic and chromosomal instability by converting into a malignant phenotype. Identifying the mechanisms that allow cells to complete chromosome duplication and segregation may thus provide a deeper understanding of tumorigenesis and ideas for therapeutic intervention.

Therefore, to understand BRCA2 tumorigenesis and potential synthetic lethal targets, it is crucial to reveal the mechanisms underlying the paradox that BRCA2 depletion causes cancer but also leads to the lethality of individuals and normal cells. Several studies have identified the dependent pathways of BRCA2-deficient tumors that have undergone malignant transformation or their resistance mechanism to PARP inhibitor treatment by screening siRNA libraries or sgRNA libraries of BRCA2-deficient tumor cells (15–18). However, these screenings are unable to replicate the intricate early stages of the transition from non-cancerous to cancerous states. Hence, there remains a necessity to unravel how BRCA2-deficient cells navigate the challenges posed by mutations, enabling their adaptation to survival pressures and facilitating the initiation of the early mechanisms driving malignant proliferation.

In this study, we conducted forward genetic synthetic viability screenings in *Caenorhabditis* *elegans* to identify the key pathways or genes that are responsible for BRCA2 deficiency-induced lethality at the organismal level. After screening more than 100,000 nematodes, we identified that NSFL1C deficiency endowed BRCA2-deficient cells with survival permission by inhibiting the stability of Aurora kinase B (AURKB) on the centromeres, which silenced the spindle assembly checkpoint (SAC) during mitosis, but did not restore the HR or replication fork stability function of BRCA2-deficient cells. Based on these studies, we propose that targeting PP2A to reactivate the SAC may be a promising strategy for treating cancer patients with *BRCA2* mutations.

## Results

### Forward genetic screenings reveal that NSFL1C loss rescues viability in BRCA2-deficient C. elegans and mammalian cells.

**Figure 1:**
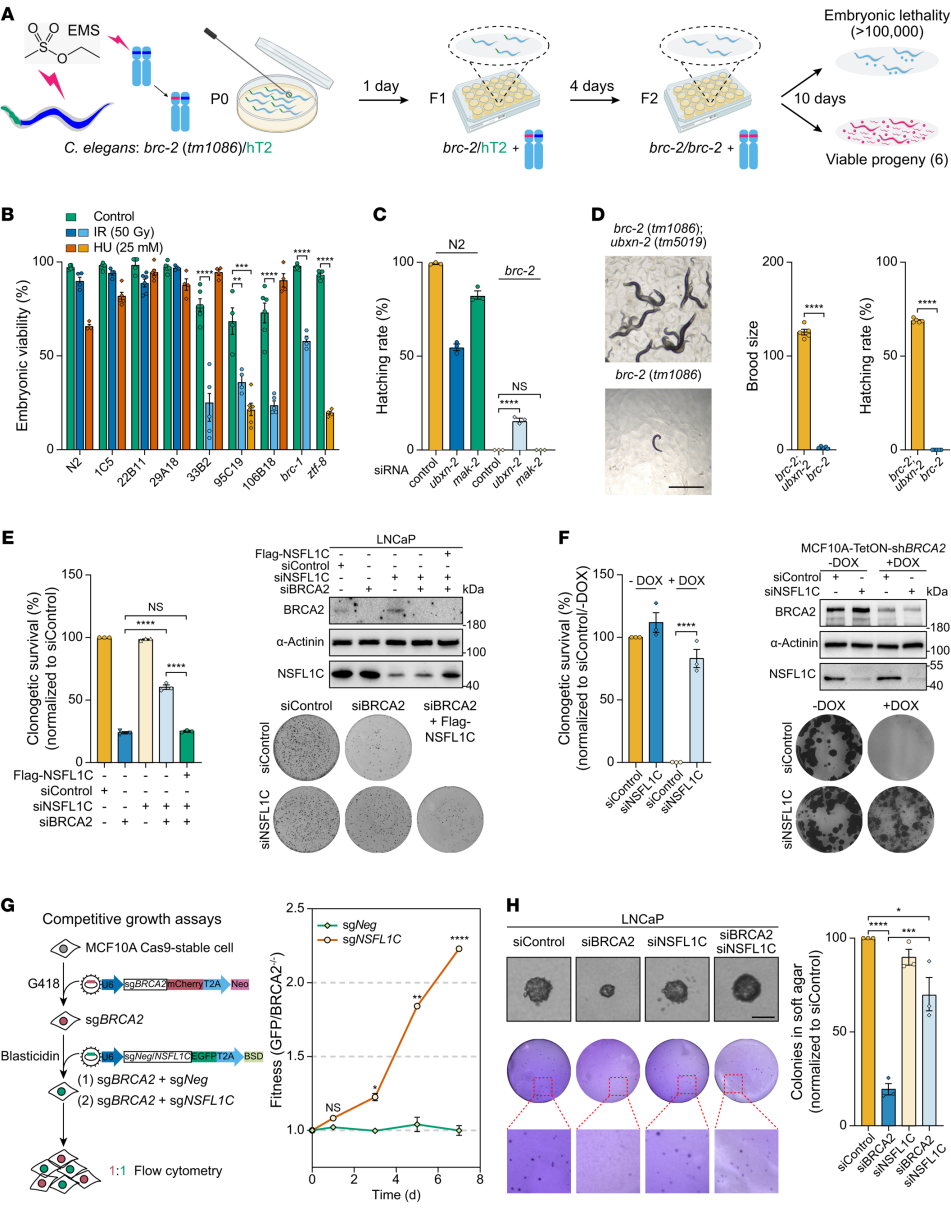
Forward genetic screenings reveal that NSFL1C loss rescues viability in BRCA2-deficient *C.* *elegans* and mammalian cells. (**A**) Schematic of screening procedure. EMS, ethyl methane sulfonate. (**B**) *C.* *elegans* strain 95C19 was sensitive to both ionizing radiation (IR) and hydroxyurea (HU). DNA damage sensitivity assays in the 6 screened *C*. *elegans* (*n* = 4 to 6). N2, wild-type strain; *brc-1*, IR-sensitive strain; *ztf-8*, HU-sensitive strain. (**C**) Deletion of *ubxn-2* prevented the lethal phenotype of *C.* *elegans* *brc-2* mutants. Hatching rate assay upon endogenous siRNA directed against *ubxn-2*/*mak-2* in the N2 and *brc-2* mutants (*n* = 3). (**D**) Deletion of *ubxn-2* restored the viability of *C.* *elegans* *brc-2* mutants. Left: Representative microscopy images. Right: Brood size and hatching rate of *brc-2*/*ubxn-2* double mutants (*n* = 5). Scale bar: 1 mm. (**E**) Clonogenic survival of LNCaP cells expressing the indicated siRNA or siRNA-resistant *NSFL1C* (*n* = 3). Immunoblotting showing BRCA2 and NSFL1C depletion in LNCaP cells. (**F**) Clonogenic survival of MCF10A-TetON-sh*BRCA2* cells expressing the indicated *NSFL1C*-targeting siRNA or transfected with control siRNA (*n* = 3). Immunoblotting showing BRCA2 and NSFL1C depletion in MCF10A cells. (**G**) Left: Schematic of the CRISPR/Cas9 system used for the competitive growth assays. Right: Competitive growth assays in MCF10A Cas9-stable cells transfected with virus expressing the indicated sgRNA (*n* = 3). (**H**) Colony growth of LNCaP cells transfected with the indicated siRNA on soft agar. Representative phase-contrast microscopy images (top left) and crystal violet staining images (bottom left). Quantitation of the number of staining colonies (*n* = 3). Scale bar: 200 μm. Data indicate the mean ± SEM. *P < 0.05, **P < 0.01, ***P < 0.001, and ****P < 0.0001. Unpaired 2-tailed Student’s *t* test was used in **D**. One-way ANOVA was used in **B**, **C**, **E**, **F**, and **H**. Two-way ANOVA was used in **G**.

To identify gene mutations that prevent embryonic death induced by the loss of BRCA2, we conducted forward genetic synthetic viability screenings in *C.* *elegans* *brc-2* (*Cebrc-2*) mutants. A total of more than 100,000 worms were evaluated, and we identified 6 worms that did not succumb to the embryonic lethality caused by *Cebrc-2* mutations (Figure 1A and Supplemental Figure 1A; supplemental material available online with this article; https://doi.org/10.1172/JCI172137DS1). By comparing these mutant strains with the homozygous *Cebrc-2*, we found that their breeding scales were similar except for 33B2 (Supplemental Figure 1B). In terms of hatching rate, 1C5, 22B11, and 29A18 reached the rate level of the wild-type strain (Supplemental Figure 1C). Among the 6 strains, only 95C19 was found to be sensitive to both ionizing radiation (IR) and hydroxyurea (HU) treatment (Figure 1B), indicating that 95C19 may not rescue the embryonic viability of *Cebrc-2* mutants by restoring HR (19) or replication fork stability (3). After sequencing the whole genome, excluding the genetic background and mutations on the same chromosome as *Cebrc-2*, we found that the mutation or the knockdown of *Ceubxn-2* significantly restored the lethality of *Cebrc-2* mutants (Figure 1C and Supplemental Figure 1, D–F). Furthermore, a *Ceubxn-2* mutant, which is a 260 bp deletion mutation across exon 1 to exon 3, could also rescue the viability of *Cebrc-2* mutants (Figure 1D and Supplemental Figure 1G). Collectively, these findings clearly showed that loss of *Ceubxn-2* rescued embryonic viability in *Cebrc-2* mutants.

Next, we verified whether the depletion of NSFL1C, the mammalian ortholog of CeUBXN-2, was beneficial to the proliferation of BRCA2-deficient cells using human cell culture experiments. First, we successfully constructed and obtained *BRCA2* and *NSFL1C* double-knockout (DKO) cells in HeLa, HCT116, and U2OS cell lines, but did not obtain any *BRCA2* single-knockout cell lines (Supplemental Figure 1H). Concordantly, reintroduction of single-guide RNA–resistant (sgRNA-resistant) *NSFL1C* into *BRCA2*/*NSFL1C* DKO cells dramatically decreased their viability (Supplemental Figure 1I). The rescue phenotype of NSFL1C depletion was also verified in the LNCaP and MCF10A-TetON *BRCA2*-knockdown cell lines (Figure 1, E and F). Furthermore, enhancement of proliferation of BRCA2-deficient MCF10A cells by *NSFL1C* knockdown was validated in a growth-based competition assay (Figure 1G). To further assess the contribution of NSFL1C to the tumorigenic potential of cells lacking BRCA2, the effects of NSFL1C depletion on the ability of these cells to grow in an anchorage-independent manner were analyzed. The results showed that NSFL1C loss markedly restored the impaired growth of LNCaP cells caused by BRCA2 depletion in soft agar (Figure 1H), suggesting that NSFL1C loss promoted malignant transformation in BRCA2-deficient cells. We also validated the functionality of another mammalian ortholog of CeUBXN-2, known as UBXN2B, and determined that its depletion did not restore the survival of BRCA2-deficient cells (Supplemental Figure 1J). These data show that NSFL1C loss confers survival permission and potentially promotes malignant transformation in BRCA2-deficient cells.

### SAC attenuation by NSFL1C depletion promotes growth of BRCA2-deficient cells.

**Figure 2:**
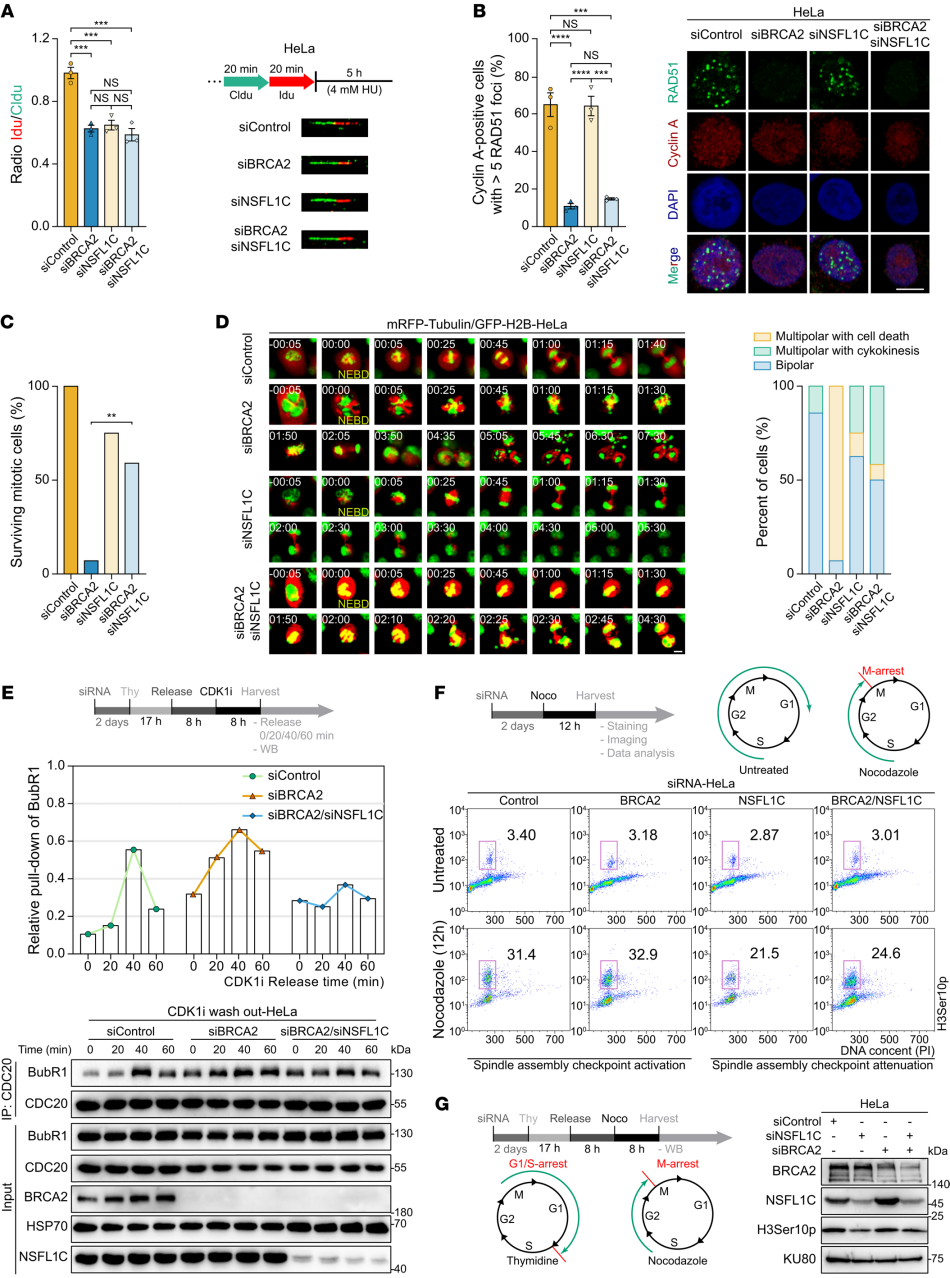
SAC attenuation by NSFL1C depletion promotes growth of BRCA2-deficient cells. (**A**) Left: Ratio of IdU versus CldU upon HU treatment (*n* = 3). Right: Schematic for labeling HeLa cells with CldU and IdU and representative images. (**B**) Quantification of IR-induced RAD51 foci in cyclin A–positive HeLa cells (left) and representative images (right) (*n* = 3). Scale bar: 10 μm. (**C** and **D**) HeLa cells expressing mRFP-tubulin and GFP-H2B were transfected with control siRNA (see Supplemental Video 1), BRCA2 siRNA (see Supplemental Video 2), NSFL1C siRNA (see Supplemental Video 3), and BRCA2/NSFL1C siRNA (see Supplemental Video 4), respectively. “NEBD” indicates the first frame after NEBD, based on the chromatin marker GFP-H2B. Times are shown in hours:minutes. Percentage of surviving mitotic cells is quantified in **C**. Representative frames are shown in **D**, left, and percentages of different fates of cells are shown in **D**, right. Scale bars: 10 μm. (**E**) Top: Schematic for synchronization experiments. Bottom: HeLa cells were treated with the indicated siRNA, synchronized by sequential thymidine-CDK1i (RO3306, 9 μM) treatment, and added to fresh medium before CDC20 immunoprecipitation. Middle: Quantitation of BubR1 relative pull-down was performed using ImageJ software. (**F**) Top: Schematic for synchronization experiments. Bottom: Flow cytometric analysis. HeLa cells were treated with the indicated siRNA, synchronized by nocodazole (Noco) treatment, and subsequently stained with H3Ser10p antibody, which is used as a marker for mitosis and propidium iodide (PI). (**G**) Left: Schematic for synchronization experiments. Right: HeLa cells were treated with the indicated siRNA and synchronized by sequential thymidine-nocodazole treatment, and cells were collected for H3Ser10p immunoblotting detection, which is used as a marker for mitosis. Data indicate the mean ± SEM. **P < 0.01, ***P < 0.001, and ****P < 0.0001. One-way ANOVA was used in **A** and **B**; χ^2^ test was used in **C**.

BRCA2 has important functions of repairing DNA damage and maintaining genome stability (2), so we first evaluated whether NSFL1C depletion reduced the spontaneous DNA damage and drug sensitivity caused by BRCA2-deficient cells. Surprisingly, NSFL1C depletion neither reduced the spontaneous DNA damage of BRCA2-deficient cells (Supplemental Figure 2, A and E) nor antagonized the sensitivity of BRCA2-deficient cells to multiple DNA damage drugs, such as cisplatin, camptothecin, and HU (Supplemental Figure 2, B and F). Next, we further tested 2 previously described functions of BRCA2 (1): protection of stalled replication forks and facilitation of HR through recruitment of RAD51 to sites of DNA breaks. First, the ability of *BRCA2*/*NSFL1C* double-silenced cells to protect stalled replication forks was still significantly weakened according to the results of a DNA fiber analysis (Figure 2A and Supplemental Figure 2E). It was noteworthy that NSFL1C deficiency could also lead to instability of replication forks. According to a reported study (20), the activation of DNA-PK can induce phosphorylation of NSFL1C. Hence, we speculated that NSFL1C, functioning as a downstream element of DNA-PK, may maintain replication fork stability in the presence of cellular damage. Second, IR-induced recruitment of RAD51 foci was still absent in *BRCA2*/*NSFL1C* double-silenced cells (Figure 2B and Supplemental Figure 2E) and *BRCA2*/*NSFL1C* DKO cells (Supplemental Figure 2C). Furthermore, using a DR-GFP assay, we found that co-depletion of NSFL1C did not reverse the HR defect caused by BRCA2 depletion (Supplemental Figure 2D). These data clearly show that the effect of NSFL1C depletion on BRCA2-deficient cells does not restore the functions of BRCA2-mediated replication fork stability maintenance or HR repair in the S/G__2__ phase. As reported, BRCA2-deficient cells progress into mitosis with incompletely replicated DNA (21), and proteins such as MUS81 contribute to the survival of BRCA2-deficient cells through promoting mitotic DNA synthesis (MiDAS) (22). To investigate this possibility, we assessed the mitotic EdU foci, a marker used to identify MiDAS as reported (21). In alignment with prior research (21), BRCA2 deficiency amplified the occurrence of mitotic cells displaying EdU foci (Supplemental Figure 2G). However, the simultaneous depletion of NSFL1C yielded no discernible impact (Supplemental Figure 2G), suggesting that the depletion of NSFL1C does not rescue the survival of BRCA2-deficient cells by promoting MiDAS.

In addition to S/G__2__ phase, BRCA2 also plays an important role in mitosis (1, 4, 5), so we further tested whether NSFL1C depletion affects BRCA2-mediated mitotic functions such as chromosome segregation. Consistent with a reported study (5), the examined *BRCA2*-silenced cells had prolonged metaphase-like arrest, and the spindles assembled in these cells were highly unstable, collapsed, and reassembled into multipolar spindles (Figure 2, C and D, and Supplemental Figure 2H). We also found that after *BRCA2*-silenced cells entered mitosis, only 7% of cells could complete mitosis, while the remaining 93% underwent apoptosis or mitotic catastrophe (Figure 2, C and D). Notably, silencing of *NSFL1C* allowed more than 50% of *BRCA2*-silenced cells to complete cell division (Figure 2, C and D), suggesting that *NSFL1C* knockdown suppresses SAC activation caused by BRCA2 depletion.

Therefore, we further tested whether NSFL1C plays a role in SAC activation to prevent the mitotic separation failure caused by BRCA2 depletion. We measured SAC strength by quantifying the amount of BubR1 that coimmunoprecipitates with CDC20. As a crucial constituent of the SAC, BubR1 operates by binding to CDC20, thereby impeding anaphase-promoting complex-mediated degradation of cyclin B1 and securin, which prevents advance to anaphase (23). After CDK1 inhibitor (CDK1i) washout, CDC20-BubR1 interaction steadily increased for 40 minutes and subsequently decreased in control cells (Supplemental Figure 2J). Interestingly, in *NSFL1C*-silenced cells, the interaction of CDC20 and BubR1 was less pronounced (Supplemental Figure 2J). Compared with *NSFL1C*-silenced cells, *BRCA2*-silenced cells exhibited a more pronounced interaction of CDC20 with BubR1, suggesting activation of the SAC due to chromosome segregation failure, and *BRCA2*/*NSFL1C* double-silenced cells exhibited attenuated SAC activation (Figure 2E). The effect of NSFL1C on the SAC was also determined under mitotic arrest caused by poison treatment. Compared with the control cells, *NSFL1C*-silenced cells exhibited reduced levels of H3Ser10p after low-dose nocodazole treatment, which is used as a marker for mitosis (24), suggesting that NSFL1C depletion can lead to the completion of mitosis due to the reduction of SAC activation upon mitotic stress (Figure 2, F and G, and Supplemental Figure 2I). Importantly, NSFL1C depletion can facilitate the successful mitosis of BRCA2-deficient cells under mitotic stress by suppressing the SAC.

Unlike BRCA2 deficiency, cells with HR defects such as BRCA1 and ATM deficiency display attenuated SAC activity (25–27). We not only validated the outcomes by quantifying the amount of BubR1 that coimmunoprecipitates with CDC20 (Supplemental Figure 2K) but also provided confirmation that the depletion of NSFL1C did not restore the viability of HeLa cells with BRCA1 or ATM deficiency (Supplemental Figure 2L). These data show that attenuated SAC activation caused by NSFL1C depletion promotes the division and survival of BRCA2-deficient cells, but this mechanism does not extend to cells lacking other HR-related factors.

### Loss of NSFL1C restabilizes kinetochore-microtubule attachments in BRCA2-deficient cells.

**Figure 3:**
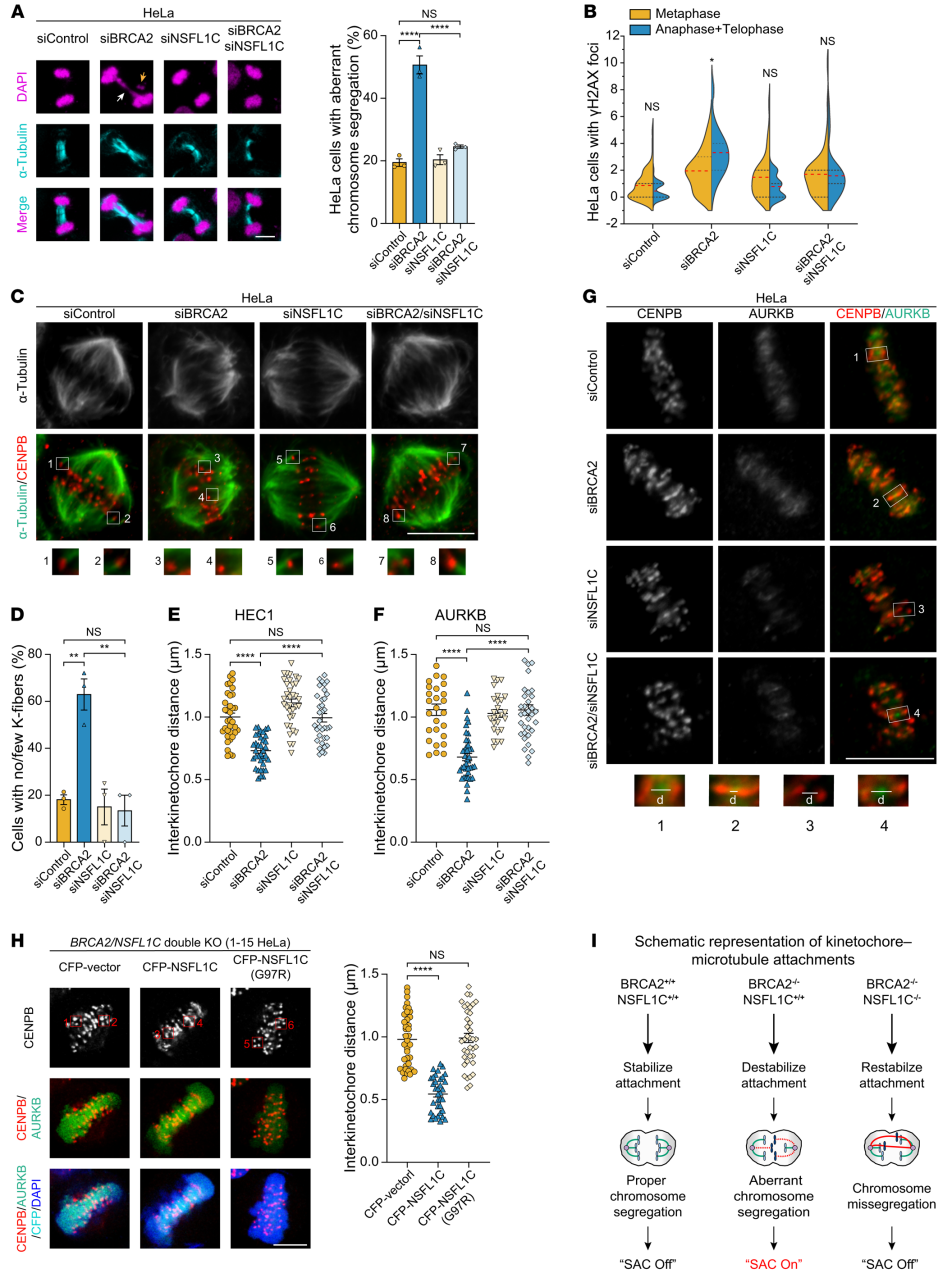
Loss of NSFL1C restabilizes kinetochore-microtubule attachments in BRCA2-deficient cells. (**A**) Loss of NSFL1C reduced aberrant chromosome segregation of BRCA2-deficient HeLa cells (*n* = 3). The white arrow points to the chromosome bridges, and the orange arrow points to the lagging chromosomes. Scale bar: 10 μm. (**B**) Violin chart of γH2AX foci in metaphase and anaphase plus telophase HeLa cells. See Supplemental Figure 3B for representative images. (**C** and **D**) Loss of NSFL1C restabilized cold-stable microtubules in BRCA2-deficient HeLa cells. (**C**) Representative images of cold-stable microtubules in cells transfected with the indicated siRNA. Cells were costained with α-tubulin and CENPB as markers for centromeres. Insets show one enlargement of the outlined regions. Scale bar: 10 μm. (**D**) Frequency of K-fiber defects (*n* = 3). (**E**–**G**) Loss of NSFL1C restored the kinetochore-microtubule attachments in BRCA2-deficient cells. HEC1, an inner kinetochore protein, are used as markers for locating centromeres, while CENPB serves as marker for centromeres. Representative images for **E** can be seen in Supplemental Figure 3E. Representative images for **F** can be seen in **G**. Insets show one enlargement of the outlined regions, and “d” represents the distance between centromeres. Scale bar: 10 μm. (**H**) Measurement of kinetochore-microtubule attachments in *BRCA2*/*NSFL1C* DKO HeLa cells expressing the sgRNA-resistant *NSFL1C* or transfected with control vector or NSFL1C-G97R mutant (95C19 *C.* *elegans* homologous mutation). Scale bar: 10 μm. (**I**) Schematic representation of kinetochore-microtubule attachments. Data indicate the mean ± SEM. *P < 0.05, **P < 0.01, and ****P < 0.0001. Unpaired, 2-tailed Student’s *t* test was used in **B**. One-way ANOVA was used in **A**, **D**, **E**, **F**, and **H**.

The SAC halts anaphase progression until all kinetochores have obtained bipolar, stable attachments to the mitotic spindle (28). BRCA2 forms complexes with PLK1, BubR1, and PP2A, and promotes PLK1-mediated phosphorylation of BubR1 (5), thereby facilitating kinetochore-microtubule attachments (K-fibers) (29, 30). Thus, BRCA2-deficient cells have unstable K-fibers, misaligned chromosomes, faulty chromosome segregation, and persistent SAC activation, as do BubR1- and PP2A-depleted cells (5, 31, 32). In subsequent experiments, we found that *BRCA2*-silenced cells produced more aberrant chromosome segregation, and depletion of NSFL1C reduced the generation of chromosome bridges and lagging chromosomes (Figure 3A and Supplemental Figure 3, A and C). In addition, more γH2AX foci were found at microtubule-chromatin junctions in *BRCA2*-silenced cells, suggesting destabilization of K-fibers, and this phenotype was attenuated in *BRCA2*/*NSFL1C* double-silenced cells (Figure 3B and Supplemental Figure 3, B and C). Next, to further confirm whether this defect in alignment was due to the impaired stability of K-fibers as previously reported (29, 31), we examined the presence of cold-stable microtubules in *BRCA2*-silenced cells. The results showed that control metaphase cells exhibited relatively intact bipolar spindles with most CENPB-stained kinetochores attached to microtubules (α-tubulin) (Figure 3, C and D, and Supplemental Figure 3D). In stark contrast, almost all the K-fibers were lost in *BRCA2*-silenced cells upon cold treatment, while *BRCA2*/*NSFL1C* double-silenced cells had more stable kinetochore-microtubule attachments (Figure 3, C and D). We also evaluated the stability of K-fibers by measuring the distance between centromeres on the metaphase chromosomes as previously reported (33). Consistent with the microtubule stability assay, *NSFL1C* knockdown restored the distance between centromeres in *BRCA2*-silenced cells (Figure 3, E–G, and Supplemental Figure 3, D and E). Under nocodazole treatment, which interferes with microtubule polymerization, the centromeres of *NSFL1C*-silenced cells (including the double-silenced cells) remained distant from each other and maintained more stable K-fibers than those of the control and *BRCA2*-silenced cells (Supplemental Figure 3, F and G). Reintroduction of sgRNA-resistant *NSFL1C* into *BRCA2*/*NSFL1C* DKO cells shortened the distance between centromeres, reflecting the instability of K-fibers, but the NSFL1C-G97R mutant (95C19 *C.* *elegans* homologous mutation) had no effect (Figure 3H). We conclude that the loss of NSFL1C allows BRCA2-deficient cells to maintain relatively stable K-fibers to inactivate the SAC and complete cell division (Figure 3I and Supplemental Figure 3H).

### NSFL1C prevents premature dissociation of AURKB from the centromeres by decreasing polyubiquitination of AURKB.

**Figure 4:**
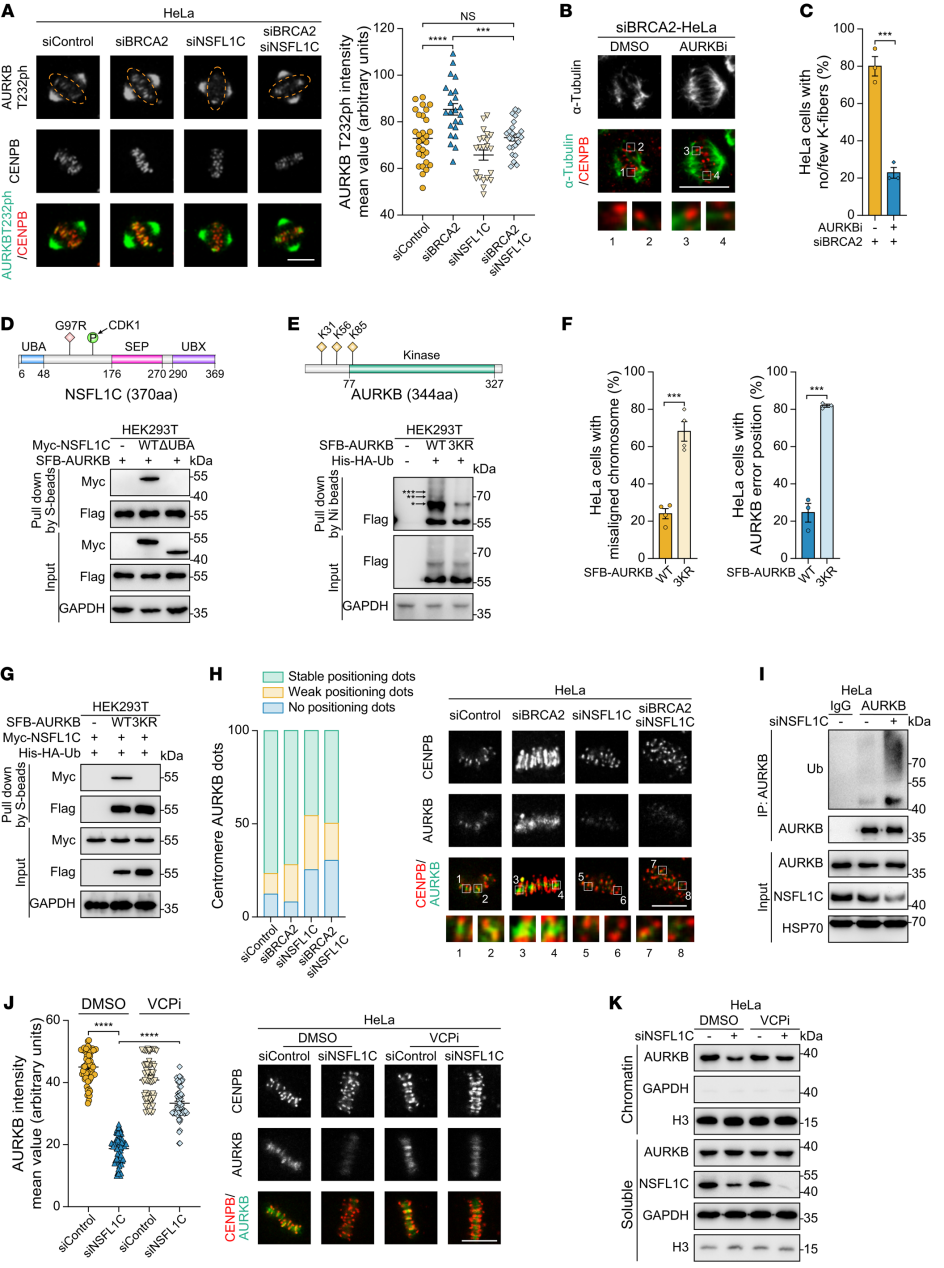
NSFL1C prevents premature dissociation of AURKB from the centromeres by decreasing polyubiquitination of AURKB. (**A**) Loss of NSFL1C restored the AURKB-T232ph intensity in BRCA2-deficient HeLa cells, which is the direct marker of AURKB activity. Quantification of AURKB-T232ph intensity (right) and representative images (left). Scale bar: 10 μm. (**B** and **C**) AURKBi (barasertib, 10 nM) restabilized cold-stable microtubules in BRCA2-deficient HeLa cells. (**B**) Representative images of cold-stable microtubules in cells transfected with the BRCA2 siRNA. Scale bar: 10 μm. (**C**) Frequency of K-fiber defects (*n* = 3). (**D**) The UBA domain of NSFL1C was important for its interaction with AURKB. (**E**) K31, K56, and K85 mutations impaired the AURKB ubiquitination. *Monoubiquitination; **diubiquitination; ***triubiquitination. (**F**) AURKB-WT maintained centromere localization in metaphase, while AURKB-3KR did not (*n* = 3 or 4). See Supplemental Figure 4I for representative images. (**G**) K31, K56, and K85 (3KR) mutations impaired AURKB interaction with NSFL1C. (**H**) NSFL1C regulated the localization of AURKB to centromeres in metaphase. Quantification of centromeres (identified as stable CENPB puncta) with no, weak, or stable puncta of AURKB (left) and representative images (right) (*n* = 3). Scale bar: 10 μm. (**I**) NSFL1C regulated ubiquitination of AURKB. (**J**) VCPi (NMS-873, 10 μM) rescued the accumulation of AURKB on the equatorial plate in *NSFL1C*-knockdown HeLa cells (*n* = 3). Scale bar: 10 μm. (**K**) VCPi (NMS-873, 10 μM) rescued the chromatin loading of AURKB in *NSFL1C*-knockdown cells. HeLa cells were treated with the indicated siRNA, synchronized by sequential nocodazole-VCPi treatment, and collected for immunoblotting detection. Data indicate the mean ± SEM. ***P < 0.001 and ****P < 0.0001. Unpaired, 2-tailed Student’s *t* test was used in **C** and **F**. One-way ANOVA was used in **A**. Two-way ANOVA was used in **J**.

BRCA2 maintains the stability of K-fibers by forming a trimeric complex with BubR1 and PP2A, while the decline of phosphorylated BubR1 (p-BubR1) and PP2A at the centromeres of BRCA2-deficient cells is considered to be one reason for the instability of K-fibers (5). Therefore, we tested whether NSFL1C deficiency could rescue centromeric p-BubR1 or PP2A levels in BRCA2-deficient cells, and found that the co-depletion of NSFL1C could neither reverse the p-BubR1 level defect caused by *BRCA2* knockdown (Supplemental Figure 4A) nor reverse the PP2A-B56 level defect (Supplemental Figure 4B). To further identify the key factors for restoring the stability of K-fibers in BRCA2 and NSFL1C double-deficient cells, the proteins related to BRCA2 and NSFL1C were purified by tandem affinity purification (TAP) and analyzed by mass spectrometry (MS). Consistent with previously reported studies, BRCA2-BubR1-PLK1 formed a tight trimeric complex in vivo (Supplemental Figure 4C). Surprisingly, AURKB, which destabilizes K-fibers to correct erroneous kinetochore-microtubule attachments via phosphorylation of the KMN network (28), was highly enriched in both MS results (Supplemental Figure 4C). Moreover, BRCA2 and NSFL1C co-located with AURKB during mitotic anaphase (Supplemental Figure 4, D and E). Therefore, we wondered whether the activity of AURKB during mitosis is regulated by BRCA2 and NSFL1C. First, we measured and quantified the level of AURKB-T232ph near CENPB, which is the direct marker of AURKB activity (34). Interestingly, the AURKB activity of the *BRCA2*-silenced cells was increased, while the AURKB activity of the *BRCA2*/*NSFL1C* double-silenced cells decreased sharply and was closer to the control cell levels (Figure 4A). The above results suggest that NSFL1C reduction may restore the stability of K-fibers in BRCA2-deficient cells by inhibiting the activity of AURKB, so direct inhibition of AURKB should have the same effect. Our subsequent experiments confirmed that treatment with a specific chemical inhibitor of AURKB (AURKBi) (35) sufficiently restored the stability of K-fibers in *BRCA2*-silenced cells (Figure 4, B and C). Together, these data suggest that NSFL1C loss reduces abnormal cell division in *BRCA2*-silenced cells by attenuating AURKB hyperactivation that leads to unstable K-fibers, thereby inhibiting SAC activation and promoting cell cycle progression.

Next, to further explore how NSFL1C regulates the activity of AURKB, we first verified the results by MS and found that NSFL1C interacted with ubiquitin-modified AURKB (Supplemental Figure 4F). NSFL1C has been reported to recognize monoubiquitinated proteins through its UBA domain and then regulate the function of binding proteins (36, 37). We also found that the NSFL1C mutant lacking the UBA domain could not interact with AURKB (Figure 4D). Therefore, we further identified the monoubiquitination sites of AURKB through MS analysis, and found that ubiquitination occurred at 3 lysine residues: K31, K56, and K85 (Supplemental Figure 4G). To verify the MS result, a pull-down experiment of AURKB under denatured conditions showed that the amount of monoubiquitinized AURKB was slightly reduced in the cells expressing the AURKB-K31R, -K56R, or -K85R mutants, and more reduced in the cells expressing the AURKB-3KR mutants (K31R/K56R/K85R) (Figure 4E and Supplemental Figure 4H). Importantly, AURKB-WT maintained centromere localization in the metaphase, while the AURKB-3KR mutant had diffuse distribution (Figure 4F and Supplemental Figure 4I), suggesting that effective AURKB monoubiquitination is necessary for its centromeric localization. The AURKB-3KR mutant had a lower level of AURKB-T232ph (Supplemental Figure 4J), suggesting that effective AURKB monoubiquitination is necessary for its function. Consistent with the previous results, NSFL1C bound to AURKB-WT, but not to the AURKB-3KR mutant, which further proved that NSFL1C interacted with monoubiquitinated AURKB through its UBA domain (Figure 4G). The above data show that monoubiquitination of AURKB is required for its centromere localization and function, and NSFL1C recognizes monoubiquitinated AURKB through its UBA domain to regulate its function.

Since NSFL1C depletion can inhibit AURKB activity in BRCA2-deficient cells, we further explored how NSFL1C regulates AURKB activity. First, we found that NSFL1C depletion decreased the accumulation of AURKB on the equatorial plate, almost completely reversing the overaccumulation of AURKB on the equatorial plate of BRCA2-deficient cells (Figure 4H). We further found that the knockdown of *NSFL1C* reduced the level of AURKB in the chromatin fraction during metaphase (Supplemental Figure 4K), suggesting that NSFL1C can promote the stable existence of AURKB at the centromeres. To further confirm whether NSFL1C affects its stability by regulating the polyubiquitination level of AURKB, we performed an in vivo ubiquitylation assay to evaluate changes in its ubiquitination. The results showed that in the cells ectopically expressing NSFL1C-WT, the polyubiquitination of AURKB was reduced, while the heterotopic expression of the NSFL1C-UBA mutant was not changed (Supplemental Figure 4L), which showed that NSFL1C could influence its ubiquitination by interacting with AURKB. In addition, after knockdown of *NSFL1C*, the polyubiquitination level of AURKB was increased (Figure 4I), and NSFL1C-mediated regulation of AURKB was independent of BRCA2 loss (Supplemental Figure 4M). This further indicates that NSFL1C may affect the stability of its protein at the centromeres by regulating the ubiquitination level of AURKB, thus controlling its activity.

NSFL1C functions as a cofactor for VCP, a ubiquitin-dependent ATPase that plays a pivotal role in maintaining cellular homeostasis through the ubiquitin-proteasome pathway (38). The monoubiquitination and deubiquitination processes become essential for postmitotic fusion of the Golgi apparatus membrane, and VCP/NSFL1C plays an important role in the deubiquitination process (36). Notably, this process differs from protein degradation mediated by proteasomes. Skillfully reversing ubiquitination, the deubiquitinase VCIP135 mediated by VCP/NSFL1C takes charge of orchestrating a seamless remodeling of the Golgi membrane (36, 39). Additionally, it has been documented that, following mitotic completion, VCP along with its cofactors UFD1/NPL4 collaboratively removes AURKB from chromatin, facilitating chromatin decondensation and the formation of the nuclear envelope (40). Therefore, we explored whether NSFL1C depletion causes VCP to extract ubiquitinated AURKB from chromatin in advance. Our results showed that treatment with a specific chemical inhibitor of VCP (VCPi) sufficiently restored the accumulation of AURKB on the equatorial plate and the stability of protein at the centromeres in *NSFL1C*-silenced cells (Figure 4J), as well as the level of AURKB in chromatin (Figure 4K).

Overall, these data show that the monoubiquitination of AURKB promotes the interaction between AURKB and NSFL1C, and thus ensures the protein stability of centromeric AURKB by decreasing the polyubiquitination of AURKB. The depletion of NSFL1C promotes the extraction of ubiquitinated AURKB from the chromatin in advance by VCP to balance the hyperactivated AURKB in BRCA2-deficient cells.

### NSFL1C promotes USP9X-mediated deubiquitination of AURKB to stabilize centromeric AURKB.

**Figure 5:**
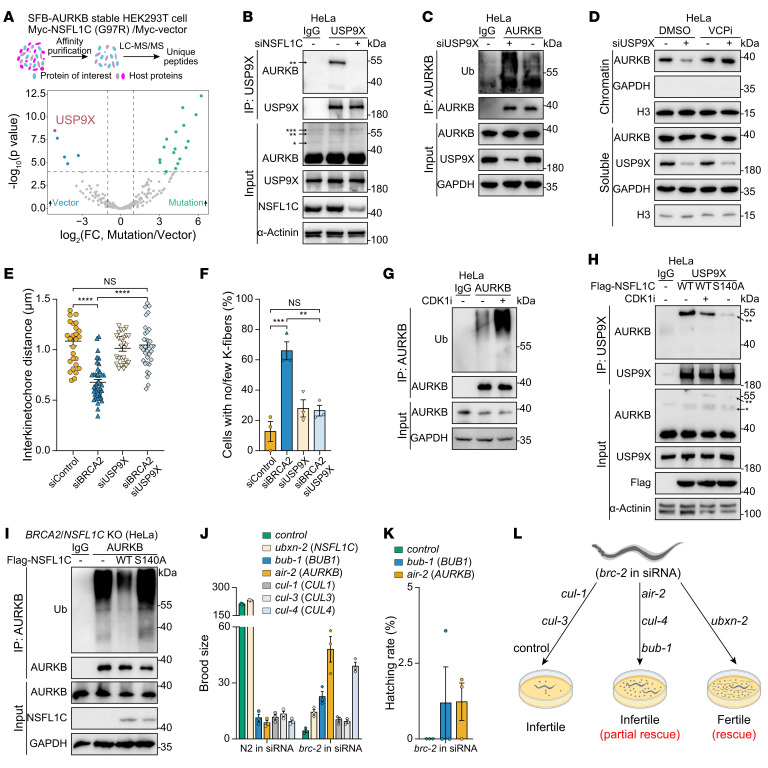
NSFL1C promotes USP9X-mediated deubiquitination of AURKB to stabilize centromeric AURKB. (**A**) Volcano plot illustrating the differentially expressed proteins (DEPs) in SFB-AURKB stable HEK293T cells transfected with Myc-NSFL1C (G97R) or empty vector. DEP analysis in the non-repetitive group used the edgeR package (https://bioconductor.org/packages/edgeR), and the volcano plot was drawn using the ggplot2 package (https://ggplot2.tidyverse.org). See Supplemental Table 3 for details. (**B**) The NSFL1C protein was required for the USP9X-AURKB interaction. HeLa cells were treated with the indicated siRNA. *Monoubiquitination; **diubiquitination; ***triubiquitination. (**C**) USP9X regulated ubiquitination of AURKB. HeLa cells were treated with the indicated siRNA. (**D**) VCPi (NMS-873, 10 μM) rescued the chromatin loading of AURKB in *USP9X*-knockdown cells. HeLa cells were treated with the indicated siRNA, synchronized by sequential nocodazole-VCPi treatment, and collected for immunoblotting detection. (**E**) Loss of USP9X restored the kinetochore-microtubule attachments in BRCA2-deficient HeLa cells. (**F**) Loss of USP9X restabilized cold-stable microtubules in BRCA2-deficient HeLa cells (*n* = 3). (**G**) CDK1 regulated ubiquitination of AURKB. HeLa cells were synchronized by CDK1i (RO3306, 9 μM) treatment and collected for immunoblotting detection. (**H**) Phosphorylation of NSFL1C by CDK1 was necessary for the USP9X-AURKB interaction. *Monoubiquitination; **diubiquitination. (**I**) Reintroduction of NSFL1C-WT rescued the increase in AURKB polyubiquitination in *BRCA2*/*NSFL1C* DKO HeLa cells, but NSFL1C-S140A had no effect. (**J**) Brood size assay upon treatment with endogenous siRNA directed against the indicated genes in the N2 and *C.* *elegans brc-2* mutants (*n* = 3). (**K**) Deletion of *air-2* and *bub-1* partially restored the viability of *C.* *elegans* *brc-2* mutants (*n* = 3). (**L**) Schematic of the effect of silencing different genes on *C.* *elegans* *brc-2* mutants. Data indicate the mean ± SEM. **P < 0.01, ***P < 0.001, and ****P < 0.0001. One-way ANOVA was used in **E** and **F**.

Next, we explored how NSFL1C decreases the polyubiquitination modification of AURKB. NSFL1C has been reported to promote the deubiquitination of its binding protein by recruiting DUB (36). Therefore, we purified and analyzed the AURKB binding protein by TAP-MS in cells expressing the empty vector or NSFL1C-G97R mutant to identify the AURKB ubiquitination regulatory protein mediated by NSFL1C. The results showed that in the cells with ectopic expression of the NSFL1C-G97R mutant, the AURKB binding protein with the most significant decrease was the deubiquitinase USP9X (Figure 5A). We further confirmed that AURKB could not interact with USP9X upon NSFL1C depletion (Figure 5B). In addition, the polyubiquitination level of AURKB in *USP9X*-silenced cells increased obviously (Figure 5C). Moreover, the *USP9X* knockdown inhibited the distribution of AURKB in the chromatin fraction during prometaphase (Figure 5D), indicating that USP9X can stabilize the accumulation of AURKB at the centromeres. Additional study results showed that treatment with a specific chemical inhibitor of VCP sufficiently restored AURKB levels in the chromatin fraction of *USP9X*-silenced cells (Figure 5D). We further found that individual silencing of *NSFL1C* or *USP9X* increased the polyubiquitination level of AURKB, while double silencing of *NSFL1C* and *USP9X* did not further increase the polyubiquitination level of AURKB (Supplemental Figure 5A), suggesting that NSFL1C and USP9X are in the same regulatory pathway. Consistent with the effect of NSFL1C depletion, we found that the depletion of USP9X also reduced the recruitment of AURKB at the centromeres, thus restoring the distance between centromeres in BRCA2-deficient cells (Figure 5E and Supplemental Figure 5, B and C). In addition, the depletion of USP9X, similarly to the depletion of NSFL1C, also restored the stability of K-fibers in BRCA2-deficient cells (Figure 5F and Supplemental Figure 5, C and D).

We further explored the upstream signal that controls NSFL1C to affect AURKB deubiquitination during mitosis. As a component of the SAC, CDK1–cyclin B ensures accurate chromosome separation and controls the process of mitosis, which prevents premature advance to anaphase (23). It has been reported that CDK1 can phosphorylate the S140 site of NSFL1C (Supplemental Figure 5E) during mitosis, which is important for Golgi disassembly-assembly (41). Interestingly, we found that CDK1i could enhance the polyubiquitination of AURKB (Figure 5G). Moreover, the CDK1i or NSFL1C-S140A mutant disrupted the interaction between NSFL1C and AURKB, but did not affect the binding of NSFL1C with USP9X (Supplemental Figure 5F). Moreover, inhibition of CDK1-mediated phosphorylation of NSFL1C interrupted the interaction between AURKB and USP9X (Figure 5H), suggesting that CDK1 mediated the formation of the AURKB-NSFL1C-UPS9X complex through phosphorylation of NSFL1C-S140. In addition, reintroduction of sgRNA-resistant *NSFL1C* into *BRCA2*/*NSFL1C* DKO cells weakened the polyubiquitination of AURKB, but NSFL1C-S140A did not, which further proved that NSFL1C phosphorylation was important for AURKB deubiquitination (Figure 5I). In conclusion, the above results show that NSFL1C is phosphorylated by CDK1 during mitosis, and promotes the deubiquitination of AURKB by binding with USP9X, thus ensuring that AURKB maintains stability at the centromeres in the metaphase of mitosis.

The above data showed that decreasing levels of AURKB by increasing its polyubiquitination level could antagonize the hyperactivation of AURKB in BRCA2-deficient cells, which was important for rescuing the lethal phenotype of BRCA2-deficient cells. Therefore, we wanted to explore whether the silencing of *Ceair-2*, the nematode homologous gene of *AURKB*, could rescue the embryonic viability of *Cebrc-2* mutants. Interestingly, silencing *Ceair-2* (*AURKB*) and its upstream activator *Cebub-1* (*BUB1*) (42) in *Cebrc-2* mutants with embryonic lethality resulted in the production of a large number of eggs, and some of the eggs hatched into adults (Figure 5, J–L). In addition, considering previous research findings that the cullin family can mediate the centromeric localization of AURKB (43–45), we also evaluated the phenotypes of silenced cullin family homologous genes in *Cebrc-2* mutants. The results showed that silencing *Cecul-1* and *Cecul-3* had no effect on the lethal phenotype of BRCA2-deficient nematodes, but silencing *Cecul-4* partially rescued it (Figure 5, J and L, and Supplemental Figure 5G). These data show that knocking down genes that can inhibit the centromeric localization and activity of AURKB can rescue or partially rescue the viability of BRCA2-deficient organisms, as can NSFL1C depletion.

### Inhibition of PP2A could reactivate the SAC in BRCA2-deficient cells.

**Figure 6:**
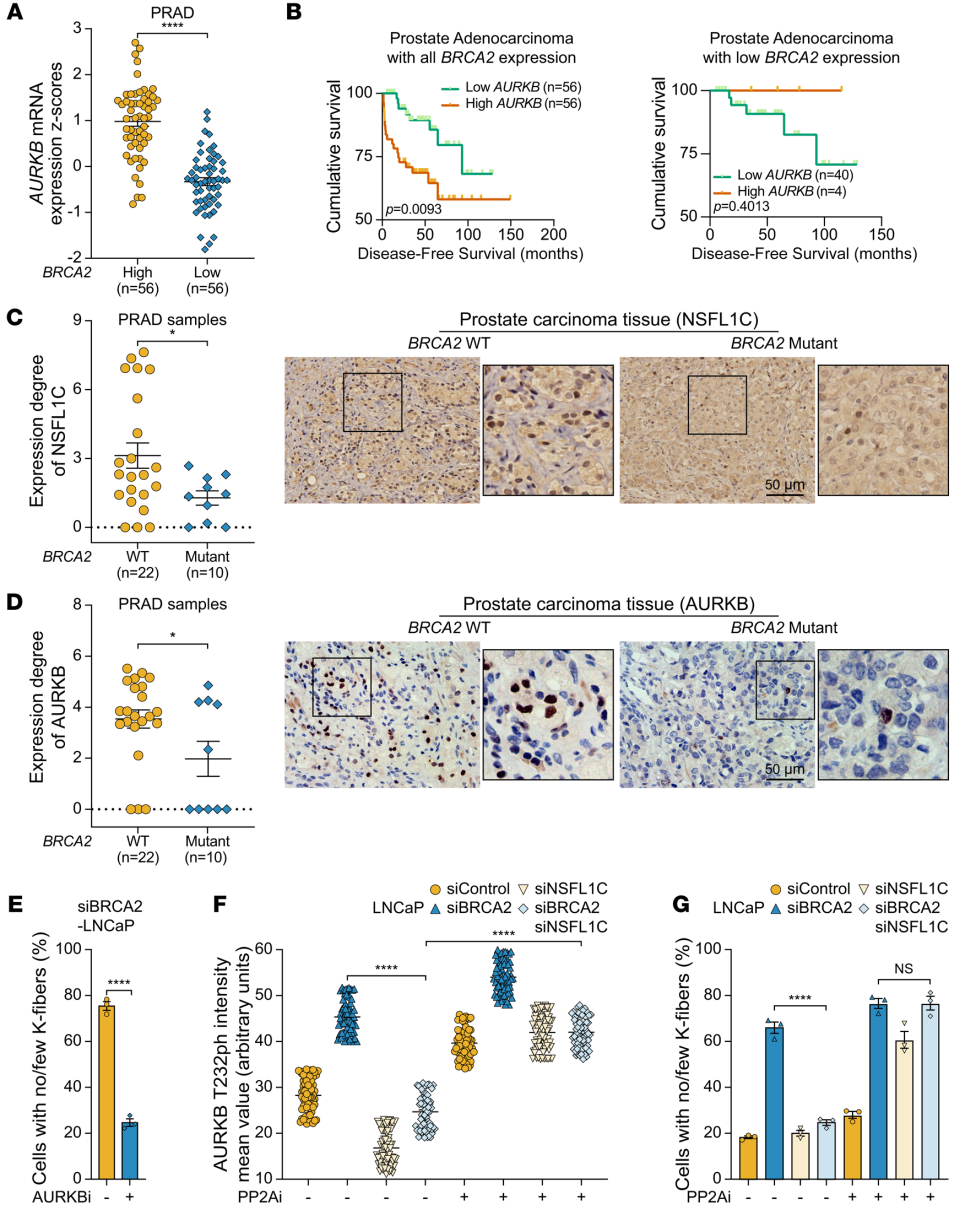
Inhibition of PP2A could reactivate the SAC in BRCA2-deficient cells. (**A**) mRNA levels of AURKB were assessed in samples from high-*BRCA2*-expression and low-*BRCA2*-expression prostate adenocarcinoma (PRAD) patients in the Memorial Sloan-Kettering Cancer Center (MSKCC) Prostate Oncogenome Project data set (from cBioPortal). Patients were separated into high *BRCA2*/*AURKB* or low *BRCA2*/*AURKB* on the basis of the 40th percentile of *BRCA2*/*AURKB* mRNA expression *z* scores. (**B**) Difference in disease-free survival between low- and all-*BRCA2*-expression PRAD patients in the MSKCC Prostate Oncogenome Project data set. Patients were separated into high *BRCA2*/*AURKB* or low *BRCA2*/*AURKB* on the basis of the 40th percentile of *BRCA2*/*AURKB* mRNA expression *z* scores. (**C** and **D**) Immunohistochemical staining of NSFL1C/AURKB was performed on tissue samples from PRAD patients with *BRCA2* WT (*n* = 22) and *BRCA2* mutant (*n* = 10). Left: Quantification of the expression degree of NSFL1C/AURKB, which was determined by the log-changed value of integral optical density. Right: Representative images. Insets show one enlargement of the outlined regions. Scale bars: 50 μm. (**E**) AURKBi (barasertib, 10 nM) restabilized cold-stable microtubules in BRCA2-deficient LNCaP cells. The frequency of K-fiber defects is shown (*n* = 3). See Supplemental Figure 6C for representative images of cold-stable microtubules. (**F**) PP2Ai (LB100, 10 μM) restored the AURKB-T232ph intensity in BRCA2/NSFL1C double-deficient LNCaP cells (*n* = 3), which is the direct marker of AURKB activity. See Supplemental Figure 6E for representative images. (**G**) PP2Ai (LB100, 10 μM) destroyed cold-stable microtubules in BRCA2/NSFL1C double-deficient LNCaP cells. The frequency of K-fiber defects is shown (*n* = 3). See Supplemental Figure 6F for representative images of cold-stable microtubules. Data indicate the mean ± SEM. *P < 0.05 and ****P < 0.0001. Unpaired, 2-tailed Student’s *t* test was used in **A** and **C**–**E**. The log-rank test was used in **B**. Two-way ANOVA was used in **F** and **G**.

Since increased AURKB polyubiquitination levels and decreased AURKB activity at the centromeres could antagonize SAC activation and rescue the viability of BRCA2-deficient individuals and cells, we questioned whether downregulated AURKB expression also exists in BRCA2-deficient tumors. First, we analyzed the mRNA levels of *AURKB* in prostate adenocarcinoma patients (46) and breast invasive ductal carcinoma patients (47) with low mRNA levels of *BRCA2* from cBioPortal (48). The results showed that the average mRNA levels of *AURKB* in BRCA2-deficient patient samples were significantly lower than those in BRCA2-proficient patient samples, especially in BRCA2-deficient prostate cancer patient samples (Figure 6A and Supplemental Figure 6A). Next, we further analyzed whether *AURKB* mRNA levels affect the prognosis of prostate cancer patients depending on BRCA2 status. Initially, we found that in all samples irrespective of BRCA2 status, patients with high *AURKB* mRNA levels had a significantly worse prognosis (Figure 6B). This aligns with previous reports suggesting a direct association between AURKB expression and the malignancy of prostate cancer, impacting prostate cell proliferation (49). However, an opposite trend was found in the subset of samples with BRCA2 defects, where patients with low *AURKB* mRNA levels tended to exhibit shorter disease-free survival (Figure 6B). This suggests that the downregulation of AURKB contributes specifically to the development of BRCA2-deficient tumors, resembling observations in nematodes and cells with BRCA2 defects.

To further determine the correlation between *BRCA2* mutations and AURKB expression in prostate cancer patients, we collected samples from prostate cancer patients with intact and mutant *BRCA2*, and analyzed the AURKB level using immunohistochemistry. Consistent with the results in the database, compared with the AURKB protein level of tumor samples from cancer patients with intact *BRCA2*, the AURKB protein level of the tumor samples from cancer patients with mutant *BRCA2* was decreased, and the same results were obtained for immunohistochemistry of NSFL1C (Figure 6, C and D). These results clearly showed that the expression level of NSFL1C/AURKB in prostate tumor samples from patients with BRCA2 deficiency was reduced, suggesting that the growth restriction phenotype as a result of SAC activation by BRCA2 deficiency may be relieved by the inhibition of NSFL1C/AURKB.

In order to further investigate the role of NSFL1C/AURKB in prostate cancer, we used BRCA2-deficient prostate cancer cell line PC3M-2B4 (50) xenotransplantation tumor models to test the tumor-forming ability in vivo. Because of the lethality associated with AURKB defects, our focus was solely on investigating NSFL1C, and our findings revealed that its loss could promote the growth of prostate tumors with BRCA2 deficiency (Supplemental Figure 6B). We further substantiated in LNCaP cells that the microtubule instability resulting from BRCA2 deficiency could be effectively reversed in prostate cancer cells by use of a specific chemical inhibitor of AURKB (Figure 6E and Supplemental Figure 6C). Additionally, we found that the depletion of NSFL1C in LNCaP cells also resulted in a diminished recruitment of AURKB to the equatorial plate during mitosis, which could be reversed by a specific chemical inhibitor of VCP (Supplemental Figure 6D).

Our results reveal that reducing AURKB levels is the key to reversing the growth inhibition caused by BRCA2 deficiency–induced K-fiber instability and SAC activation, so how to reactivate the recognition of deficient K-fibers and ultimately kill BRCA2-deficient tumor cells is a very important issue. Considering that the dephosphorylation of AURKB substrates by PP2A phosphatase is the upstream factor that silences the AURKB signal (28), we wondered whether treatment with the PP2A inhibitors (PP2Ais) could reactivate AURKB in *BRCA2*/*NSFL1C* double-silenced cells, which can simulate BRCA2 defect tumor cell death as a result of SAC activation. The results showed that NSFL1C depletion inactivated AURKB activity in *BRCA2*-silenced LNCaP cells, while PP2Ai significantly restored AURKB activity in *BRCA2*/*NSFL1C* double-silenced LNCaP cells (Figure 6F and Supplemental Figure 6E). In addition, we found that after treatment with PP2Ai, the kinetochore-microtubule attachment of *BRCA2*/*NSFL1C* double-silenced LNCaP cells became as unstable as that of the *BRCA2*-silenced LNCaP cells, suggesting that the PP2Ai could reactivate the SAC (Figure 6G and Supplemental Figure 6F). We conclude that the inactivation of AURKB promotes the development of BRCA2-deficient tumors by suppressing SAC activation, while PP2Ais can reactivate the SAC.

### PP2A is an attractive synthetic lethal therapeutic target for BRCA2-mutated cancer.

**Figure 7:**
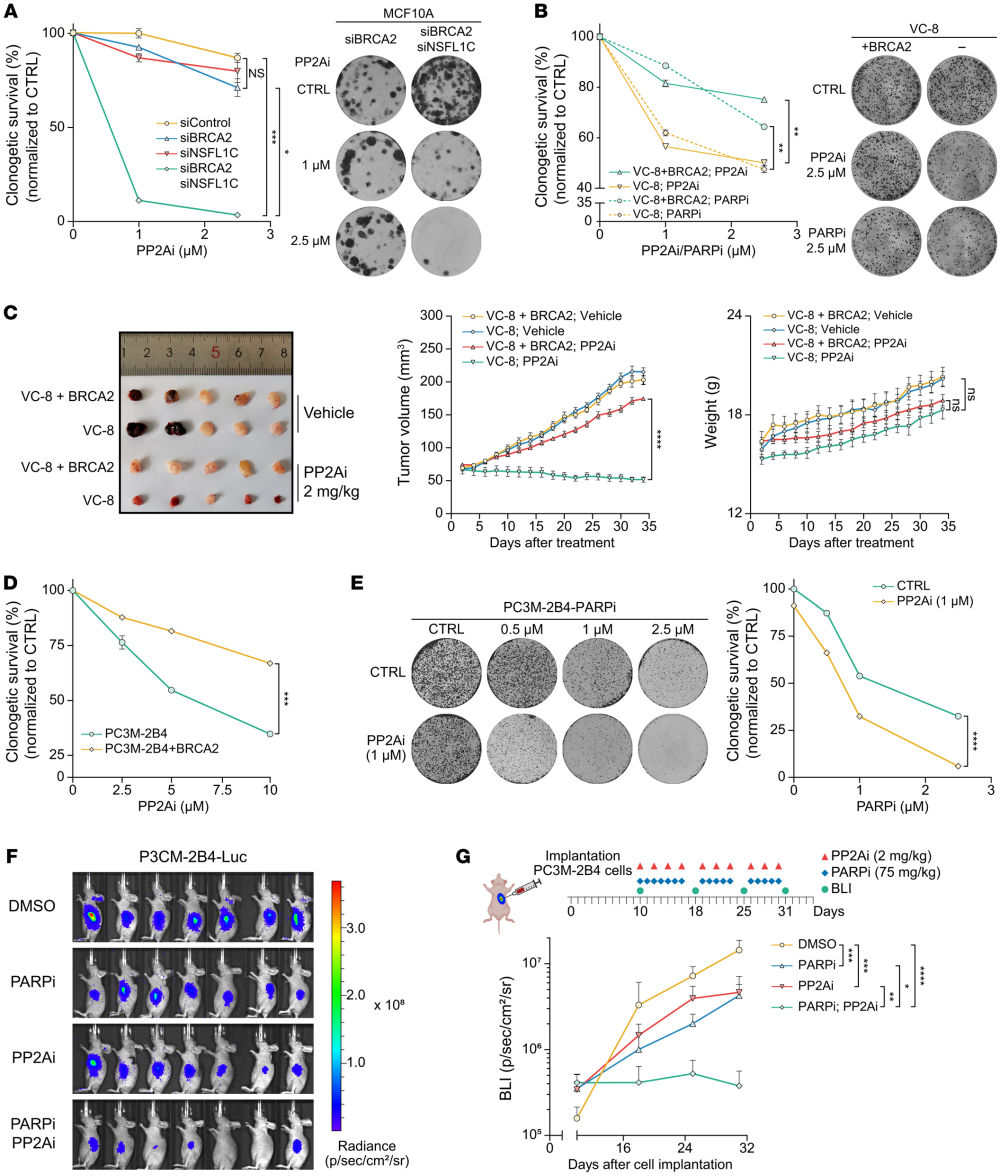
PP2A is an attractive synthetic lethal therapeutic target for *BRCA2*-mutated cancers. (**A**) MCF10A cells expressing the indicated siRNA were treated with the control or the indicated dose of PP2Ai (LB100) (24 hours), and cell survival rates were counted by calculation of the colony numbers (*n* = 3). (**B**) VC-8/VC-8 plus BRCA2 cells were treated with the control, indicated dose of PARPi (olaparib), or the indicated dose of PP2Ai (LB100) (24 hours), and cell survival rates were counted by calculation of the colony numbers (*n* = 3). (**C**) Defects in BRCA2 enhanced tumor regression induced by PP2Ai (LB100) treatment. VC-8 or VC-8 plus BRCA2 cells were used in a xenograft tumor assay with PP2Ai (LB100) treatment (*n* = 10 per group). (**D**) PC3M-2B4 cells transfected with the indicated plasmid were treated with the control or the indicated dose of PP2Ai (LB100) (24 hours), and cell survival rates were counted by calculation of the colony numbers (*n* = 3). (**E**) PP2Ai (LB100) and PARPi (olaparib) have synergistic therapeutic effects. PC3M-2B4 cells were treated with the control, indicated dose of PP2Ai (LB100), or the indicated dose of PARPi (olaparib) (24 hours), and cell survival rates were counted by calculation of the colony numbers (*n* = 3). (**F** and **G**) Representative images (31 days after implantation) and schematic for the xenograft tumor assay. PC3M-2B4 cells expressing stable luciferase were established and implanted in nude mice. The mice were randomized into 4 treatment groups (*n* = 7 or 8 per group) 10 days after implantation. The bioluminescent imaging levels were acquired every 7 days using an IVIS Spectrum CT imaging system. Data indicate the mean ± SEM. *P < 0.05, **P < 0.01, ***P < 0.001, and ****P < 0.0001. Two-way ANOVA was used in **A**–**E** and **G**.

Our previous results show that BRCA2-deficient cells promote tumor development by inactivating the SAC, and that PP2Ais can reactivate the SAC. As a result, PP2A may be an exciting potential synthetic lethal target for BRCA2-deficient tumor cells. To test whether PP2Ais can specifically kill BRCA2-deficient cells that have an inactivated SAC, we determined the colony formation ability of *BRCA2*-silenced cells that activate the SAC and *BRCA2*/*NSFL1C* double-silenced cells that silence the SAC under treatment with PP2Ai. Surprisingly, PP2Ai had a weak effect on control cells and SAC-activated *BRCA2*-silenced cells, but strongly killed SAC-silenced *BRCA2*/*NSFL1C* double-silenced cells (Figure 7A and Supplemental Figure 7A). As evidenced by a decrease in H3Ser10p levels, effective SAC activation could not be achieved by BRCA2-deficient VC-8 cells compared with the same VC-8 cells reconstituted with wild-type BRCA2 after low-dose nocodazole treatment (Supplemental Figure 7C), so they are an optimal cell line to test whether PP2Ais are effective against BRCA2-deficient and SAC-inactivated cells. We examined the impact of BRCA2 deficiency on PP2Ai sensitivity in the BRCA2-deficient VC-8 cell line and a derivative that was reconstituted with wild-type BRCA2. Excitingly, the PP2Ai had a strong killing effect on SAC-silenced BRCA2-deficient VC-8 cells, but had little effect on SAC-activated VC-8 cells reconstituted with BRCA2 (Supplemental Figure 7, B and D). Moreover, the sensitivity of VC-8 cells lacking BRCA2 to PP2Ai was similar to their sensitivity to a PARP inhibitor (PARPi) (Figure 7B), a drug that has been widely used in clinical practice to treat patients with *BRCA2* mutations (51). Furthermore, when using VC-8 cell or VC-8 plus BRCA2 cell xenotransplantation tumor models to test sensitivity to the PP2Ai in vivo, we found that the VC-8 tumors lacking BRCA2 were more sensitive to PP2Ai treatment than those expressing wild-type BRCA2 (Figure 7C).

BRCA2 is the most frequently mutated DNA damage repair factor in prostate cancer patients, with a high mutation rate of 12.7% in advanced prostate cancer patients (52). Prostate cancer cells with BRCA2 deficiency may tolerate the genomic and chromosomal instability caused by BRCA2 deficiency by silencing SAC activation in the process of cancer occurrence and progression. Therefore, we further tested PP2Ai sensitivity in the BRCA2-deficient prostate cancer cell line PC3M-2B4 (50). In comparison with the exogenous BRCA2 overexpression group (Supplemental Figure 7E), PC3M-2B4 cells in the blank control group exhibited a heightened sensitivity to PP2Ai (Figure 7D and Supplemental Figure 7F). In addition, the *BRCA2*/*NSFL1C* double-silenced group in LNCaP cells that silence the SAC also showed higher sensitivity compared with the control group and SAC-activated *BRCA2*-silenced cells (Supplemental Figure 7A). These data show that synthetic lethality can be achieved in BRCA2-deficient prostate tumor cells by reactivation of the SAC with PP2Ai, suggesting that PP2A is an attractive synthetic lethal therapeutic target for prostate cancer patients with *BRCA2* mutations.

PARPis cause the stagnation and collapse of DNA replication forks through PARP trapping, thus relying on HR repair factors such as BRCA2 to repair DNA double-strand breaks (51). Therefore, BRCA1, BRCA2, and other BRCA-like defective cells have strong sensitivity to PARPis. Since PARPis and PP2Ais target the 2 different mechanisms of DNA damage repair defects and SAC activation silencing in BRCA2-deficient tumor cells, we speculated that the combined use of PARPi and PP2Ai should have prominent advantages.

First, we found that BRCA2-deficient cells with silenced SAC activation were resistant to PARPi, suggesting that PARPi could activate the SAC in M phase by causing a large amount of DNA damage (Supplemental Figure 7, G and H). Moreover, the PP2Ai strongly increased the sensitivity of BRCA2-deficient prostate cells with silenced SAC activation to PARPi (Supplemental Figure 7H). Importantly, the BRCA2-deficient prostate cell line PC3M-2B4 had excellent sensitivity to combined treatment with PARPi and PP2Ai (Figure 7E). To better evaluate the efficacy of combined PARPi and PP2Ai therapy in vivo, we implanted PC3M-2B4 cells stably expressing luciferase into nude mice, and evaluated the effect of a single inhibitor and combination therapy on tumors. Consistent with the above results, the PARPi and PP2Ai had excellent synergistic effects on killing of tumor cells (Figure 7, F and G, and Supplemental Figure 7I). In conclusion, these results show that PP2A is a synthetic lethal target for BRCA2-deficient tumor cells, and the PP2Ais reduce potential drug resistance to PARPis and enhance their therapeutic effect.

## Discussion

Depletion or mutation of the *BRCA2* gene leads to serious genomic and chromosomal instability, which is harmful to cell growth and organismal development. How cancer cells with BRCA2 deficiency can endure these pressures and malignantly proliferate remains to be elucidated. In this study, we identified through genetic screenings that mutation of the *NSFL1C* homologous gene *Ceubxn-2* could reverse the lethal phenotype of BRCA2-deficient nematode offspring, and further revealed that NSFL1C depletion facilitated BRCA2-deficient cell survival permission by suppressing activation of the spindle assembly checkpoint (SAC). In the case of BRCA2-deficient cells, the continuously activated Aurora kinase B (AURKB) activated the SAC and caused cell death, while *NSFL1C* knockdown promoted VCP to extract AURKB from the centromeres and suppressed SAC activity. Moreover, SAC inactivation is common in BRCA2-deficient prostate cancer patients and many BRCA2-deficient cell lines, and PP2A inhibitors (PP2Ais) that can restore SAC activity are potential drugs for the synthetic lethality of *BRCA2*-mutated cancer cells.

Previous studies have found that the embryonic viability of *Brca1*-deficient mice can be rescued by the loss of *53BP1* (53), laying the foundation for the later discovery that the destruction of the 53BP1/RIF1/Shieldin axis (54–56) can restore homologous recombination (HR) repair. Although *PTIP* and *PARP1* deletions have been identified in BRCA2-deficient embryonic stem cells (ESCs) as rescuing the viability of cells (12, 13), no gene that is able to rescue BRCA2 deficiency at the organismal level has been found. Since the BRCA2 protein is highly conserved (57), we tried to carry out unbiased forward genetic synthetic viability screenings on the *Cebrc-2* mutants. We found that *Ceubxn-2* mutation and large-fragment deletion could rescue the embryonic viability of *Cebrc-2* mutants, and depletion of the mammalian homologous gene *NSFL1C* could also prevent the BRCA2-deficient cell death phenotype.

We found that the *Ceubxn-2* mutation did not restore sensitivity to replication pressure drugs or ionizing radiation treatment in the *Cebrc-2* mutants. Furthermore, loss of NSFL1C, the mammalian ortholog of CeUBXN-2, could not restore the stability of the BRCA2-deficient cell replication fork or HR repair efficiency, which showed that NSFL1C regulated BRCA2-mediated function through independent replication fork protection and HR repair pathways. There were still a large number of γH2AX signals and abnormal chromosomes in the prometaphase of BRCA2/NSFL1C double-deficient mammalian cells (Figure 3B and Supplemental Figure 3B), suggesting that NSFL1C modulation is different from the mitotic DNA synthesis (MiDAS) mediated by RAD52 (58), mitotic end joining mediated by polymerase θ (TMEJ) (59, 60), and chromosomal stability maintenance mediated by CIP2A-TOPBP1 during mitosis (16).

In addition to replication fork protection and HR repair, BRCA2 also participates in important events of mitosis, such as SAC activation (1). BRCA2 depletion destroys the stability of the genome by damaging the replication fork and decreasing the efficiency of HR in the S/G__2__ phase of the cell cycle. Fortunately, BRCA2-deficient cells strongly activate the SAC, which prevents these cells with unstable genomes from completing mitosis. The dual surveillance mechanisms of BRCA2 in both the S/G__2__ and M phases minimize the malignant transformation of cells. Our results reveal that organisms or cells with BRCA2 depletion can survive as long as SAC activation is suppressed, even if the problem of genomic instability is not solved. This finding may explain why BRCA2-deficient tumor cells isolated from patients have defects in the stability of their replication forks and decreased efficiency of HR, but the SAC is suppressed. Therefore, PP2Ai, which can reactivate the SAC, can kill BRCA2-deficient tumor cells very effectively, making it a potential therapeutic drug for cancer patients with *BRCA2* mutations. In addition, since PP2Ais kill tumor cells through different mechanisms compared with DNA damage drugs that target HR defects, such as PARPis, we speculate that PP2Ais may be able to remedy tumor cells’ acquired drug resistance.

PP2A is recognized as a tumor suppressor, and previous research has underscored the potential of its activators in treating castration-resistant prostate cancer (61). We speculate that PP2A activators, in addition to their role in reducing AR protein levels (61), may also exert their effects by suppressing AURKB activity through breaking the balance of these competitive reversible phosphorylation events at the kinetochore (28). This hypothesis arises from the notable overexpression of AURKB in prostate cancer, which is directly associated with the aggressiveness of the disease (49, 62). It is intriguing to note that our research has revealed that PP2Ais also exhibit cytotoxic effects on prostate tumors. However, these effects are predominantly observed in tumors characterized by BRCA2 deficiency, primarily achieved through the reactivation of the SAC. This indicates that the influence of PP2A on prostate tumor growth might be contingent on the status of BRCA2. Consequently, it is important to consider the mutation status of *BRCA2* before deciding to use PP2A activators or inhibitors to treat prostate cancer patients.

In conclusion, we found that depleting NSFL1C or silencing the SAC pathway could prevent BRCA2 lethal defects, and explored how BRCA2-deficient organisms or cells could tolerate severe genomic and chromosome instability, providing a mechanism for the development of BRCA2-deficient tumors. We also explained in detail that NSFL1C could prevent SAC inactivation caused by the premature dissociation of AURKB from the centromeres by reducing the polyubiquitination of AURKB. Our research suggests that PP2A inhibition to reactivate the SAC can be an attractive synthetic lethal agent for the treatment of cancer patients with *BRCA2* mutations.

## Methods

An expanded Methods section is available as Supplemental Methods.

### C. elegans maintenance and strains.

Worms were grown at 20°C on nematode growth media (NGM) plates seeded with the bacterial *E. coli* strain OP50 as a food source according to standard protocols and methods (63, 64). The N2 Bristol strain served as the wild type. Mutants 1C5, 22B11, 29A18, 33B2, 95C19, and 106B18 were obtained by ethyl methane sulfonate (EMS; MilliporeSigma, M0880) mutagenesis. *brc-2* (*tm1086*)/ht2 and *brc-1* (*tm1145*) were provided by the Caenorhabditis Genetics Center (Minneapolis, Minnesota, USA); *ztf-8* (*tm2176*) and *ubxn-2* (*tm5019*) were provided by the National BioResource Project (Tokyo, Japan); and *brc-2* (*tm1086*)/*ubxn-2* (*tm5019*) was generated in our laboratory.

### Forward genetic screenings.

A total of 10,000 synchronized *brc-2* (*tm1086*)/ht2 L4 larvae were washed 3 times with M9 buffer (20 mM KH__2__PO__4__, 40 mM Na__2__HPO__4__, 80 mM NaCl, and 1 mM MgSO__4__) and subsequently incubated with 50 mM EMS while shaking for 4 hours at room temperature. Furthermore, the worms were washed 4 times with M9 buffer and recovered at room temperature for a half hour on NGM plates. After that, *brc-2* (*tm1086*)/ht2 mutants with optimal vitality were selected to produce offspring for 3 consecutive days. In the F__1__ generation, *brc-2* (*tm1086*) heterozygous mutant progeny with GFP fluorescence continued to be selected, while in the F__2__ generation, *brc-2* (*tm1086*) homozygous mutant progeny without GFP fluorescence were selected. One week later, the worms were observed for the production of offspring.

### C. elegans RNAi treatments.

RNAi constructs were transformed into HT115 bacteria, and the transformants were cultured overnight in Luria-Bertani liquid medium containing 100 μg/mL ampicillin (MilliporeSigma, A9518) at 37°C with vigorous shaking and then densely plated onto NGM plates containing 100 μg/mL carbenicillin and 4 mM isopropyl thiogalactoside (MilliporeSigma, I6758) (65). The plates were allowed to dry before use. For the RNAi inheritance assays, F__0__ L4 larvae were added to the RNAi NGM plate for breeding, and then the F__1__ L4 larvae were added to a new RNAi NGM plate for brood size and hatching rate observations.

### DNA damage sensitivity assays in C. elegans.

To detect sensitivity to DNA damage, the following method was used (66). Worms were bleached and synchronized to the L4 stage. They were incubated for an additional 18–20 hours, and then the young adult worms were ready for further analysis. Age-matched young adults were transferred to OP50-seeded plates for ionizing radiation (IR) exposure and hydroxyurea-containing (HU-containing) seeded plates for the HU sensitivity assay, and then incubated for 18–20 hours at 20°C. After that, the worms were transferred to NGM plates and incubated for 3 hours at 20°C. The worms were separated (5 per plate) and incubated at 20°C for 4 hours; parent (P0) worms were removed and discarded. After incubation at 20°C for 20–24 hours, the numbers of unhatched/dead eggs and hatched F__1__ worms were counted.

### Cell culture and synchronization.

HEK293T, HeLa, HCT116, U2OS, and MCF10A cells were obtained from the American Type Culture Collection. DR-GFP reporter U2OS, VC-8, and VC-8 plus BRCA2 cells were a gift from Jun Huang (Zhejiang University, Hangzhou, China). mRFP-tubulin/GFP-H2B HeLa cells were a gift from Genze Shao (Peking University, Beijing, China). LNCaP cells were a gift from Yuke Chen (Peking University First Hospital). PC3M-2B4 cells were a gift from Haoyun Liu (Peking University). HEK293T, HCT116, U2OS, VC-8, VC-8 plus BRCA2, HeLa, and mRFP-tubulin/GFP-H2B HeLa cells were cultured in DMEM (Thermo Fisher Scientific, C11995500BT) supplemented with 10% FBS and 1% penicillin/streptomycin (Macgene, CC004). PC3M-2B4 and LNCaP cells were cultured in RPMI 1640 (Macgene, CM10040) supplemented with 10% FBS and 1% penicillin/streptomycin. MCF10A cells were cultured in F12 (Macgene, CM10092) supplemented with 5% horse serum, 20 ng/mL epidermal growth factor (Macgene, CC102), 0.5 mg/mL hydrocortisone (Macgene, CC103), 100 ng/mL cholera toxin (Macgene, CC104), 10 μg/mL insulin (Macgene, CC101), and 1% penicillin/streptomycin. All cell lines were maintained at 37°C in a 5% CO__2__ incubator.

For synchronization of cells in mitosis, 100–200 ng/mL nocodazole (TargetMol, T2802) was added to the growth media, and the cells were cultured for 12 hours before harvesting. For synchronization by double thymidine block, the cells were treated with 2 mM thymidine (TargetMol, TWP2911) for 17 hours, released for 8 hours, and then subjected to a second thymidine (2 mM) treatment for 15 hours.

### Competitive growth assays.

MCF10A Cas9-stable cells were transduced with virus particles expressing U6-mCherry-sg*BRCA2*. Twenty-four hours after transfection, the virally transduced cells were selected using 750 μg/mL of G418 (Yeasen, 60220ES03). One day after infection, sg*BRCA2*-expressing cells were transduced with virus particles expressing U6-sg*Neg*-EGFP or U6-sg*NSFL1C*-EGFP. Twenty-four hours after transfection, the virally transfected cells were selected using 10 μg/mL of blasticidin (Yeasen, 60218ES10) and 750 μg/mL of G418. Two days after transfection, sg*BRCA2*- and EGFP-expressing cells (double transfection) were mixed 1:1 (3,000 cells each) and seeded in a 12-well Nest plate (Nest, 712001). The proportion of GFP-positive cells was measured by flow cytometry at 0, 1, 3, 5, and 7 days.

### Soft agar anchorage-independent growth.

For anchorage-independent growth assays, the volume ratio of the bottom well was 1.2% agar (MilliporeSigma, A5431)/2× RPMI 1640/FBS = 4.5:4.5:1, and the volume ratio of the top well was 0.8% agar/2× RPMI 1640/FBS = 4.5:4.5:1. Cells (1,000 LNCaP cells) were seeded on 6-well plates 36 hours after siRNA transfection. After 10–14 days, the colonies were captured using a microscope (SOPTOP, XD-T) equipped with a digital camera (TOUPCAM, E3ISPM12000KPA). Then the cells were washed with PBS and stained with 0.1% crystal violet (MilliporeSigma, C0775). The stained wells were washed with PBS, and the colonies were counted.

### HR efficiency assay.

For the DR-GFP homologous recombination (HR) assay (67), GFP-positive cells were detected by flow cytometry 3 days after I-SceI transfection, and the data were analyzed with FlowJo v10 software. 

### Tandem affinity purification.

HEK293T cells stably expressing SFB-AURKB or SFB-NSFL1C were lysed with NETN buffer on ice for 20 minutes. Cell lysates were centrifuged at 20,000*g* for 15 minutes at 4°C, and the supernatants were incubated with streptavidin-Sepharose beads (GE Healthcare Life Science, 17511301) for 2 hours at 4°C. The resin was washed 3 times with NETN buffer and eluted with elution buffer (2 mg/mL biotin, 20 mM Tris-HCl [pH 8.0], 100 mM NaCl, 1 mM EDTA, and 0.5% Nonidet P-40) overnight at 4°C. The eluates were combined and then incubated with S-protein agarose (Merck Millipore, 69704) for 4 hours at 4°C. The S-protein agarose beads were washed 3 times with NETN buffer. The proteins bound to the S-protein agarose beads were eluted with 40 μL of 1× SDS loading buffer, separated by SDS-PAGE, and then visualized by Coomassie blue staining. The proteins that interact with BRCA2 were obtained through endogenous immunoprecipitation experiments, as shown in Supplemental Methods. The eluted proteins were identified by mass spectrometry analysis.

### Colony formation assay.

Cells were treated with the indicated doses of IR, camptothecin (6 hours), HU (24 hours), cisplatin (24 hours), LB100 (24 hours), and olaparib (24 hours). The same number of cells exposed to the different treatments were plated in 60 mm dishes or 6-well plates. After 10–14 days, the cells were washed with PBS and stained with 0.1% Coomassie brilliant blue (10% ethylic acid plus 50% carbinol plus 40% H__2__O) for 30 minutes at room temperature. The stained dishes were washed with water, and the colonies were counted.

### Mouse models and xenografts.

All of the procedures involving mice and experimental protocols were performed in accordance with the Guidelines of the Peking University Animal Care and Use Committee (Beijing, China). BALB/c nude female mice (5 weeks) were obtained from HFK Biotechnology Co. Ltd. (Beijing, China). Xenograft experiments were conducted using cells (5 × 10^^7^^ VC-8 and VC-8 plus BRCA2, *n* = 10 per group, or 1 × 10^^7^^ PC3M-2B4, *n* = 13 shControl, 12 shNSFL1C) suspended in 100 μL PBS and 100 μL Matrigel Matrix High Concentration (Corning, 354248) for subcutaneous injection. Drugs were administered 2 weeks after tumor inoculation, and LB100 (2 mg/kg) was injected intraperitoneally every 2 days. Tumor growth was measured using a caliper every 2 days, and the tumor volume was calculated using the following formula: tumor volume = length × width^^2^^/2.

For the bioluminescence imaging (BLI) assay, we generated stable luciferase-expressing PC3M-2B4 cell lines. Xenograft experiments were conducted using 1 × 10^^7^^ PC3M-2B4-Luc cells suspended in 100 μL PBS and 100 μL Matrigel Matrix High Concentration for subcutaneous injection (*n* = 35). Tumor formation was confirmed by bioluminescence, and the mice were randomized into 4 groups after 10 days from the implantation for different treatments: DMSO (*n* = 7), olaparib (*n* = 8), LB100 (*n* = 7), and olaparib combined with LB100 (*n* = 8). One nude mouse died on the 28th day and on the 30th day after the implantation in the combination group and the olaparib single-drug group, respectively. The drugs were administered intraperitoneally according to the pattern shown in Figure 7G. The BLI levels were acquired every 7 days using an IVIS Spectrum CT imaging system (PerkinElmer). The mice were intraperitoneally injected with 200 μL of 15 mg/mL d-luciferin (PerkinElmer, 122799) and subsequently anesthetized with isoflurane inhalation and photographed 10 minutes after injection. The radiance within each area of interest was determined using Living Image Software 4.3.1 (PerkinElmer).

### Statistics.

Unless stated otherwise, Prism 8 (GraphPad Software) was used to generate graphs, perform statistical tests, and calculate *P* values. Error bars, statistical tests, and number of independent repeats (*n*) are indicated in the figure legends. Statistical tests included 2-tailed Student’s *t* test, χ^^2^^ test, log-rank test, 1-way ANOVA, and 2-way ANOVA. Quantitative data are presented as means ± SEM. *P* less than 0.05 was considered statistically significant; *P < 0.05, **P < 0.01, ***P < 0.001, ****P < 0.0001.

### *Study approval*.

Ethics approval (2023YAN035-002) was obtained from the National Unit of Clinical Trial Ethics Committee of Peking University First Hospital (Beijing, China) to use the clinical paraffin sections for research purposes. The animal studies were approved by the Peking University Animal Care and Use Committee (Beijing, China; LA2021268).

### *Data availability*.

All data are presented in the main text and supplemental material. Values for all data points in graphs are reported in the Supplemental Supporting Data Values file.

## Author contributions

Jian Wang performed a majority of the experiments. YC, MR, and WY conducted immunohistochemistry. SL and YL conducted forward genetic synthetic viability screenings. WL conducted mouse work. KC constructed plasmids. XAZ cowrote and proofread the paper. ZX, YX, XL, and WW discussed the paper. Jiadong Wang and Jian Wang initiated the study, designed experiments, analyzed data, and cowrote the paper. All authors read and approved the final manuscript.

## Supplementary Material

Supplemental data

Supplemental table 3

Supplemental video 1

Supplemental video 2

Supplemental video 3

Supplemental video 4

Supporting data values

## Version 1 11/07/2023

In-Press Preview

## Version 2 01/02/2024

Electronic publication

## References

[1] Venkitaraman AR (2014). Cancer suppression by the chromosome custodians, BRCA1 and BRCA2. Science.

[2] Roy R (2011). BRCA1 and BRCA2: different roles in a common pathway of genome protection. Nat Rev Cancer.

[3] Schlacher K (2011). Double-strand break repair-independent role for BRCA2 in blocking stalled replication fork degradation by MRE11. Cell.

[4] Choi E (2012). BRCA2 fine-tunes the spindle assembly checkpoint through reinforcement of BubR1 acetylation. Dev Cell.

[5] Ehlén Å (2020). Proper chromosome alignment depends on BRCA2 phosphorylation by PLK1. Nat Commun.

[6] Suzuki A (1997). Brca2 is required for embryonic cellular proliferation in the mouse. Genes Dev.

[7] Sharan SK (1997). Embryonic lethality and radiation hypersensitivity mediated by Rad51 in mice lacking Brca2. Nature.

[8] Ludwig T (1997). Targeted mutations of breast cancer susceptibility gene homologs in mice: lethal phenotypes of Brca1, Brca2, Brca1/Brca2, Brca1/p53, and Brca2/p53 nullizygous embryos. Genes Dev.

[9] Connor F (1997). Tumorigenesis and a DNA repair defect in mice with a truncating Brca2 mutation. Nat Genet.

[10] Friedman LS (1998). Thymic lymphomas in mice with a truncating mutation in Brca2. Cancer Res.

[11] Kuznetsov SG (2008). Mouse embryonic stem cell-based functional assay to evaluate mutations in BRCA2. Nat Med.

[12] Ray Chaudhuri A (2016). Replication fork stability confers chemoresistance in BRCA-deficient cells. Nature.

[13] Ding X (2016). Synthetic viability by BRCA2 and PARP1/ARTD1 deficiencies. Nat Commun.

[14] Lee H (1999). Mitotic checkpoint inactivation fosters transformation in cells lacking the breast cancer susceptibility gene, Brca2. Mol Cell.

[15] Fugger K (2021). Targeting the nucleotide salvage factor DNPH1 sensitizes BRCA-deficient cells to PARP inhibitors. Science.

[16] Adam S (2021). The CIP2A-TOPBP1 axis safeguards chromosome stability and is a synthetic lethal target for BRCA-mutated cancer. Nat Cancer.

[17] Guillemette S (2015). Resistance to therapy in BRCA2 mutant cells due to loss of the nucleosome remodeling factor CHD4. Genes Dev.

[18] Tang M (2021). Genome-wide CRISPR screens reveal cyclin C as synthetic survival target of BRCA2. Nucleic Acids Res.

[19] Moynahan ME (2001). BRCA2 is required for homology-directed repair of chromosomal breaks. Mol Cell.

[20] Kotula E (2013). DNA-PK target identification reveals novel links between DNA repair signaling and cytoskeletal regulation. PLoS One.

[21] Groelly FJ (2022). Mitotic DNA synthesis is caused by transcription-replication conflicts in BRCA2-deficient cells. Mol Cell.

[22] Lai X (2017). MUS81 nuclease activity is essential for replication stress tolerance and chromosome segregation in BRCA2-deficient cells. Nat Commun.

[23] Kops GJ (2005). On the road to cancer: aneuploidy and the mitotic checkpoint. Nat Rev Cancer.

[24] Hendzel MJ (1997). Mitosis-specific phosphorylation of histone H3 initiates primarily within pericentromeric heterochromatin during G2 and spreads in an ordered fashion coincident with mitotic chromosome condensation. Chromosoma.

[25] Eliezer Y (2014). Interplay between the DNA damage proteins MDC1 and ATM in the regulation of the spindle assembly checkpoint. J Biol Chem.

[26] Xiao M (2022). Dual-functional significance of ATM-mediated phosphorylation of spindle assembly checkpoint component Bub3 in mitosis and the DNA damage response. J Biol Chem.

[27] Chabalier C (2006). BRCA1 downregulation leads to premature inactivation of spindle checkpoint and confers paclitaxel resistance. Cell Cycle.

[28] Foley EA, Kapoor TM (2013). Microtubule attachment and spindle assembly checkpoint signalling at the kinetochore. Nat Rev Mol Cell Biol.

[29] Elowe S (2007). Tension-sensitive Plk1 phosphorylation on BubR1 regulates the stability of kinetochore microtubule interactions. Genes Dev.

[30] Suijkerbuijk SJ (2012). Integration of kinase and phosphatase activities by BUBR1 ensures formation of stable kinetochore-microtubule attachments. Dev Cell.

[31] Lampson MA, Kapoor TM (2005). The human mitotic checkpoint protein BubR1 regulates chromosome-spindle attachments. Nat Cell Biol.

[32] Foley EA (2011). Formation of stable attachments between kinetochores and microtubules depends on the B56-PP2A phosphatase. Nat Cell Biol.

[33] Maresca TJ, Salmon ED (2009). Intrakinetochore stretch is associated with changes in kinetochore phosphorylation and spindle assembly checkpoint activity. J Cell Biol.

[34] Yasui Y (2004). Autophosphorylation of a newly identified site of Aurora-B is indispensable for cytokinesis. J Biol Chem.

[35] Yang J (2007). AZD1152, a novel and selective aurora B kinase inhibitor, induces growth arrest, apoptosis, and sensitization for tubulin depolymerizing agent or topoisomerase II inhibitor in human acute leukemia cells in vitro and in vivo. Blood.

[36] Huang S (2016). Monoubiquitination of syntaxin 5 regulates Golgi membrane dynamics during the cell cycle. Dev Cell.

[37] Meyer HH (2002). Direct binding of ubiquitin conjugates by the mammalian p97 adaptor complexes, p47 and Ufd1-Npl4. EMBO J.

[38] Meyer H (2012). Emerging functions of the VCP/p97 AAA-ATPase in the ubiquitin system. Nat Cell Biol.

[39] Wang Y (2004). VCIP135 acts as a deubiquitinating enzyme during p97-p47-mediated reassembly of mitotic Golgi fragments. J Cell Biol.

[40] Ramadan K (2007). Cdc48/p97 promotes reformation of the nucleus by extracting the kinase Aurora B from chromatin. Nature.

[41] Uchiyama K (2003). The localization and phosphorylation of p47 are important for Golgi disassembly-assembly during the cell cycle. J Cell Biol.

[42] Kawashima SA (2010). Phosphorylation of H2A by Bub1 prevents chromosomal instability through localizing shugoshin. Science.

[43] Sumara I (2007). A Cul3-based E3 ligase removes Aurora B from mitotic chromosomes, regulating mitotic progression and completion of cytokinesis in human cells. Dev Cell.

[44] Maerki S (2009). The Cul3-KLHL21 E3 ubiquitin ligase targets aurora B to midzone microtubules in anaphase and is required for cytokinesis. J Cell Biol.

[45] Krupina K (2016). Ubiquitin receptor protein UBASH3B drives Aurora B recruitment to mitotic microtubules. Dev Cell.

[46] Taylor BS (2010). Integrative genomic profiling of human prostate cancer. Cancer Cell.

[47] Ciriello G (2015). Comprehensive molecular portraits of invasive lobular breast cancer. Cell.

[48] Cerami E (2012). The cBio cancer genomics portal: an open platform for exploring multidimensional cancer genomics data. Cancer Discov.

[49] Chieffi P (2006). Aurora B expression directly correlates with prostate cancer malignancy and influence prostate cell proliferation. Prostate.

[50] Chakraborty G (2020). Significance of BRCA2 and RB1 co-loss in aggressive prostate cancer progression. Clin Cancer Res.

[51] Lord CJ, Ashworth A (2017). PARP inhibitors: synthetic lethality in the clinic. Science.

[52] Robinson D (2015). Integrative clinical genomics of advanced prostate cancer. Cell.

[53] Bunting SF (2010). 53BP1 inhibits homologous recombination in Brca1-deficient cells by blocking resection of DNA breaks. Cell.

[54] Mirman Z (2018). 53BP1-RIF1-shieldin counteracts DSB resection through CST- and Polα-dependent fill-in. Nature.

[55] Dev H (2018). Shieldin complex promotes DNA end-joining and counters homologous recombination in BRCA1-null cells. Nat Cell Biol.

[56] Noordermeer SM (2018). The shieldin complex mediates 53BP1-dependent DNA repair. Nature.

[57] Martin JS (2005). RAD-51-dependent and -independent roles of a Caenorhabditis elegans BRCA2-related protein during DNA double-strand break repair. Mol Cell Biol.

[58] Bhowmick R (2016). RAD52 facilitates mitotic DNA synthesis following replication stress. Mol Cell.

[59] Mateos-Gomez PA (2015). Mammalian polymerase θ promotes alternative NHEJ and suppresses recombination. Nature.

[60] Ceccaldi R (2015). Homologous-recombination-deficient tumours are dependent on Polθ-mediated repair. Nature.

[61] McClinch K (2018). Small-molecule activators of protein phosphatase 2A for the treatment of castration-resistant prostate cancer. Cancer Res.

[62] Lee EC (2006). Targeting Aurora kinases for the treatment of prostate cancer. Cancer Res.

[63] Brenner S (1974). The genetics of Caenorhabditis elegans. Genetics.

[64] Stiernagle T (2006). Maintenance of C. elegans. WormBook.

[65] Timmons L, Fire A (1998). Specific interference by ingested dsRNA. Nature.

[66] Kim HM, Colaiácovo MP (2015). DNA damage sensitivity assays in Caenorhabditis elegans. Bio Protoc.

[67] Gunn A, Stark JM (2012). I-SceI-based assays to examine distinct repair outcomes of mammalian chromosomal double strand breaks. Methods Mol Biol.

