# Supplementary material for: PP2A inhibition causes synthetic lethality in BRCA2-mutated prostate cancer models via spindle assembly checkpoint reactivation
Source: J Clin Invest. 2024 Jan 2;134(1):e172137. doi: 10.1172/JCI172137 (PMC10760972; doi:10.1172/JCI172137)
Supplement: Supplemental data [file jci-134-172137-s155.pdf]

Supplementary data for

PP2A inhibition causes synthetic lethality in *BRCA2*-mutated prostate cancer models via reactivating spindle assembly checkpoint

Jian Wang *et al.*

*Corresponding author: Jiadong Wang, wangjd@bjmu.edu.cn

This PDF file includes:

Supplementary Figures 1 to 7

Supplementary materials and methods

Supplementary Tables 1 to 2

Other Supplementary data for this manuscript include the following:

Supplementary Videos 1 to 4

Supplementary Table 3

Supplemental Figure 1

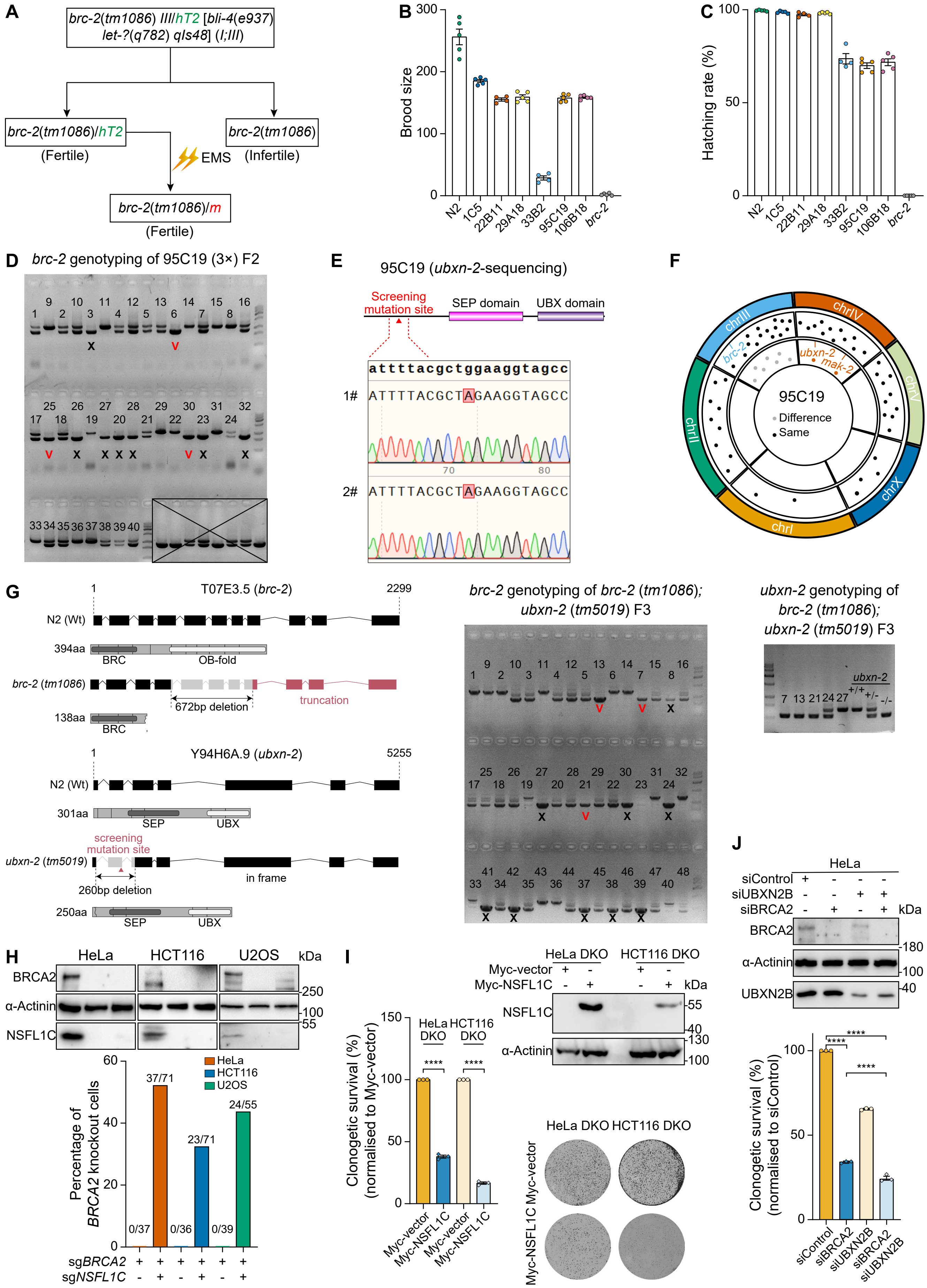

Supplemental Figure 1 (related to Figure 1). Forward genetic screenings that reveal NSFL1C loss rescues viability in BRCA2-deficient *C. elegans* and mammalian cells. (**A**) Schematic of the simple screening procedure. *m*, beneficial mutation (red mark). (**B**) Brood size of N2 and 6 screened *C. elegans* (n = 4 or 5). (**C**) Hatching rate of N2, *brc-2* and 6 screened *C. elegans* (n = 4 or 5). (**D**) *brc-2* genotyping of 95C19. X, inviable; V, viable (red mark). (**E**) 95C19 first-generation sequencing results of *ubxn-2* in screened mutation sites. (**F**) Schematic of the second-generation genome exon sequencing differences between 95C19 and N2, *brc-2*/hT2. *brc-2* is located on chromosome 3, while *brc-2* is not linked to *ubxn-2* on the chromosome, so the mutated genes on this chromosome were excluded (Supplemental Figure 1**D**). See Supplemental Table 2 for details. (**G**) Schematics of the gene structure of *brc-2* (T07E3.5) or *ubxn-2* (Y94H6A.9) (left). *brc-2* genotyping of *brc-2*; *ubxn-2* double mutant (middle). *ubxn-2* genotyping of *brc-2*; *ubxn-2* double mutant (right). (**H**) Percentage of *BRCA2* knockout cells in HeLa, HCT116 and U2OS cells expressing the indicated sgRNA. Representative immunoblotting showing BRCA2 and NSFL1C depletion in HeLa, HCT116 and U2OS cells. (**I**) Clonogenic survival of *BRCA2*/*NSFL1C* DKO HeLa/HCT116 cells expressing sgRNA-resistant *NSFL1C* or transfected with control vector. DKO double knockout (n = 3); Immunoblotting showing reintroduction of NSFL1C in *BRCA2*/*NSFL1C* DKO HeLa/HCT116 cells. (**J**) Clonogenic survival of HeLa cells expressing the indicated siRNA (n = 3). Immunoblotting showing BRCA2 and UBXN2B depletion in HeLa cells. Data indicate the mean ± SEM. Unpaired two-tailed Student's t test was used in **I**. One-way ANOVA was used in **J**.

Supplemental Figure 2

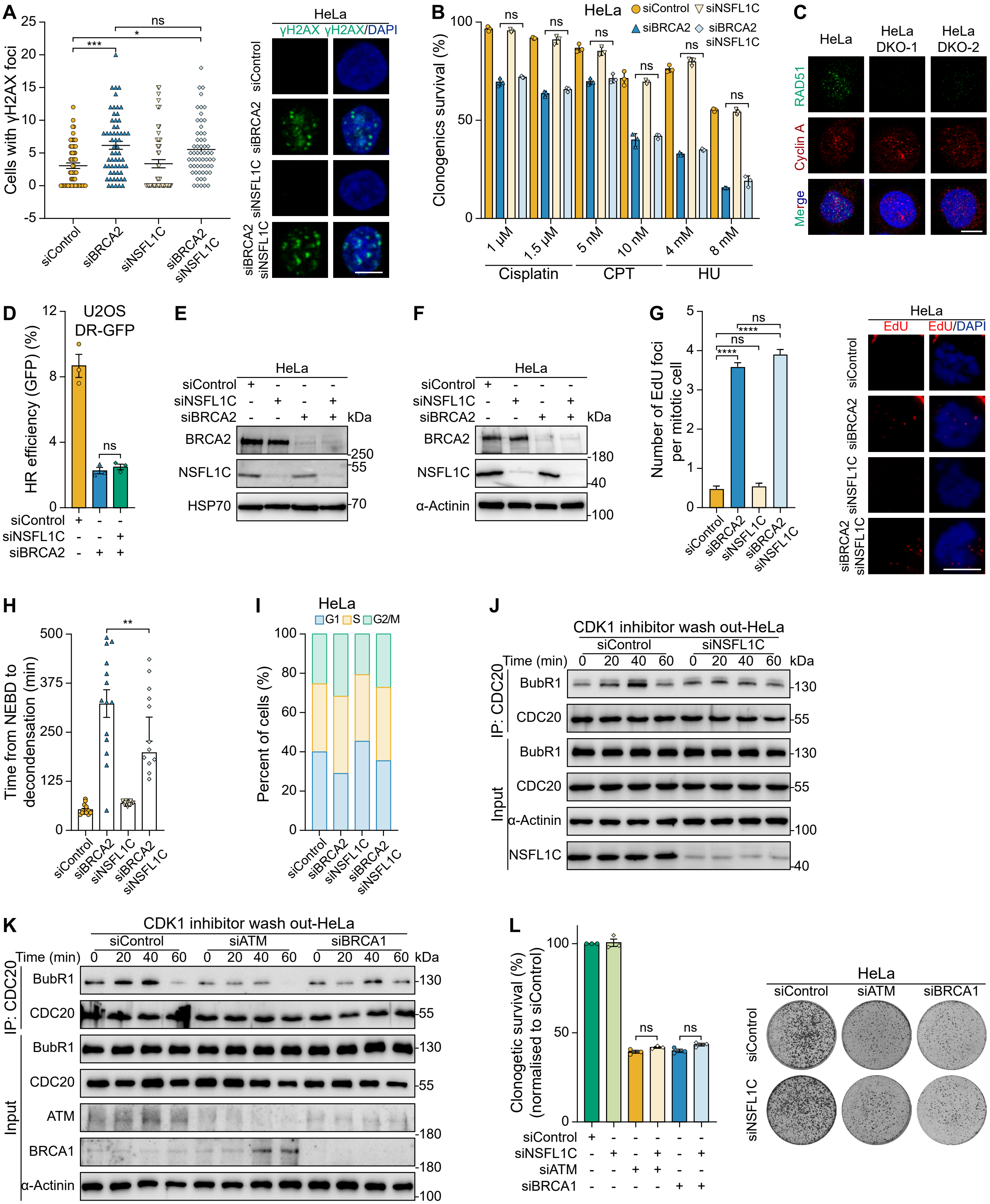

Supplemental Figure 2 (related to Figure 2). Spindle assembly checkpoint (SAC) attenuation by NSFL1C depletion promotes the growth of BRCA2-deficient cells. (**A**) Loss of NSFL1C did not restore γH2AX foci formation in interphase of BRCA2-deficient HeLa cells. Scale bar, 10 μm. (**B**) HeLa cells expressing the indicated siRNA were treated with the control or indicated dose of cisplatin, CPT, or HU, and cell survival rates were counted by calculating the colony numbers (n = 3). (**C**) Ionizing radiation (IR)-induced RAD51 foci was not restored in *BRCA2*/*NSFL1C* DKO HeLa cells. Scale bar, 10 μm. (**D**) Loss of NSFL1C did not restore HR efficiency in BRCA2-deficient U2OS cells (n = 3). (**E**) Immunoblotting showing BRCA2 and NSFL1C depletion in HeLa cells. (**F**) Immunoblotting showing BRCA2 and NSFL1C depletion in HeLa cells. (**G**) Quantification of mitotic EdU foci (left). HeLa cells were treated with the indicated siRNAs and collected 9 h after release from thymidine block with 20 mM EdU added during the last one hour. Representative images were shown (right). Scale bars, 10 μm. (**H**) Statistical analysis of the mitotic duration of HeLa cells from nuclear envelope breakdown (NEBD) to anaphase (Figure 2**D**). (**I**) Flow cytometric analysis of the cell cycle. HeLa cells were treated with the indicated siRNAs, and subsequently stained with propidium iodide (PI). (**J–K**) HeLa cells were treated with the indicated siRNA, synchronized by sequential thymidine-CDK1i (RO3306, 9 μM) treatment and added to fresh medium prior to CDC20 immunoprecipitation. (**L**) Clonogenic survival of HeLa cells expressing the indicated siRNA, and cell survival rates were counted by calculating the colony numbers (n = 3). Data indicate the mean ± SEM. One-way ANOVA was used in **A**, **B**, **D**, **G**, **H** and **L**.

Supplemental Figure 3

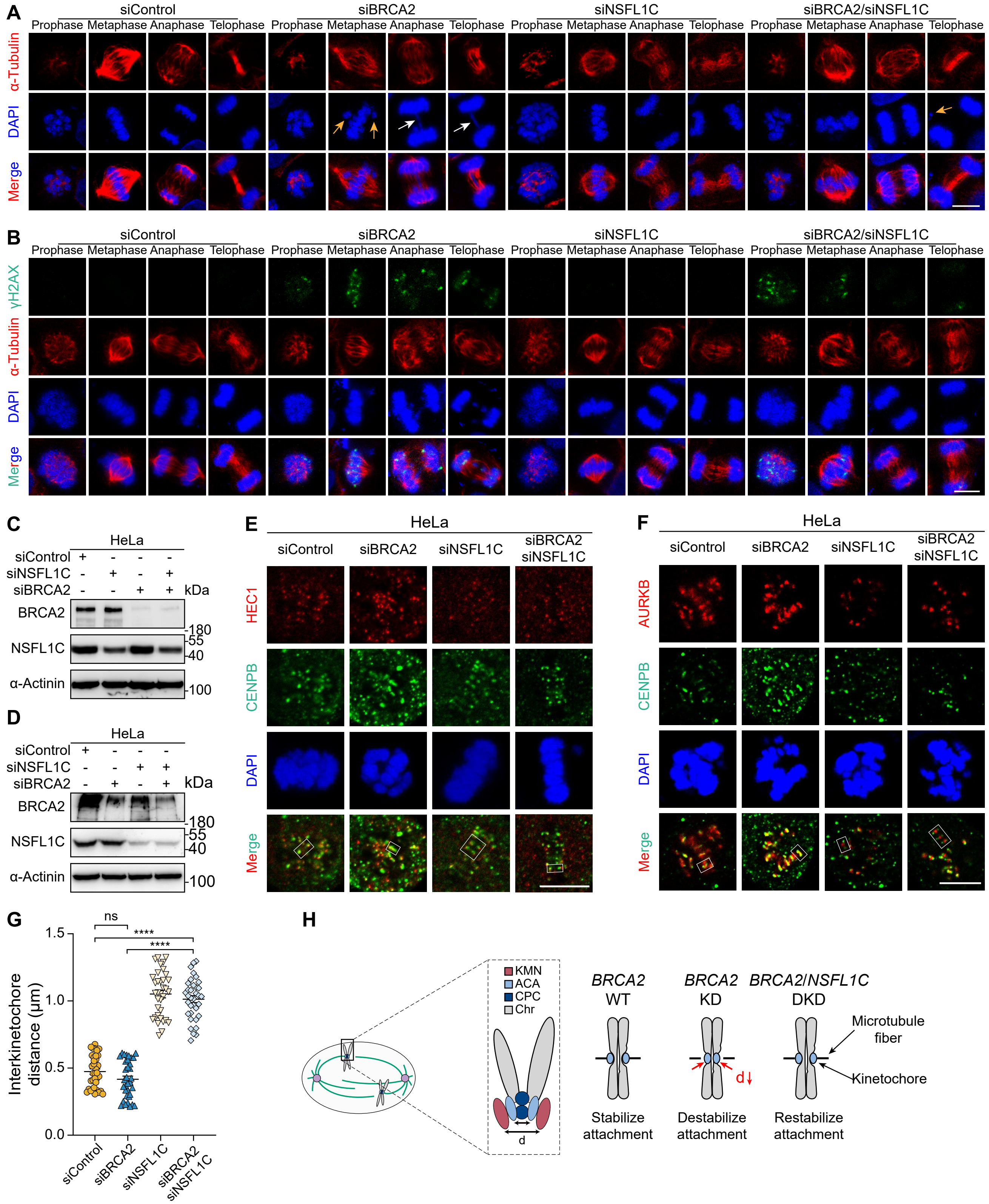

Supplemental Figure 3 (related to Figure 3). Loss of NSFL1C restabilizes kinetochore–microtubule attachments in BRCA2-deficient cells. (**A–B**) Representative images of M phase cell division and γH2AX foci. HeLa cells were treated with the indicated siRNAs, synchronized by nocodazole treatment and added to fresh medium. The white arrow points to the chromosome bridges, and the orange arrow points to the lagging chromosomes. Scale bar, 10 μm. (**C**) Immunoblotting showing BRCA2 and NSFL1C depletion in HeLa cells. (**D**) Immunoblotting showing BRCA2 and NSFL1C depletion in HeLa cells. (**E**) Representative images of Figure 3**E**. HEC1, inner kinetochore protein, as markers for locating centromeres; CENPB, as markers for centromeres. Scale bar, 10 μm. (**F–G**) Loss of NSFL1C restored the kinetochore–microtubule attachments in BRCA2-deficient HeLa cells upon nocodazole treatment in prophase. AURKB, centromeric intermediate protein, as markers for locating centromeres; CENPB, as markers for centromeres. Scale bar, 10 μm. (**H**) Schematic representation of kinetochore stretching and kinetochore–microtubule attachments. Data indicate the mean ± SEM. One-way ANOVA was used in **G**.

Supplemental Figure 4

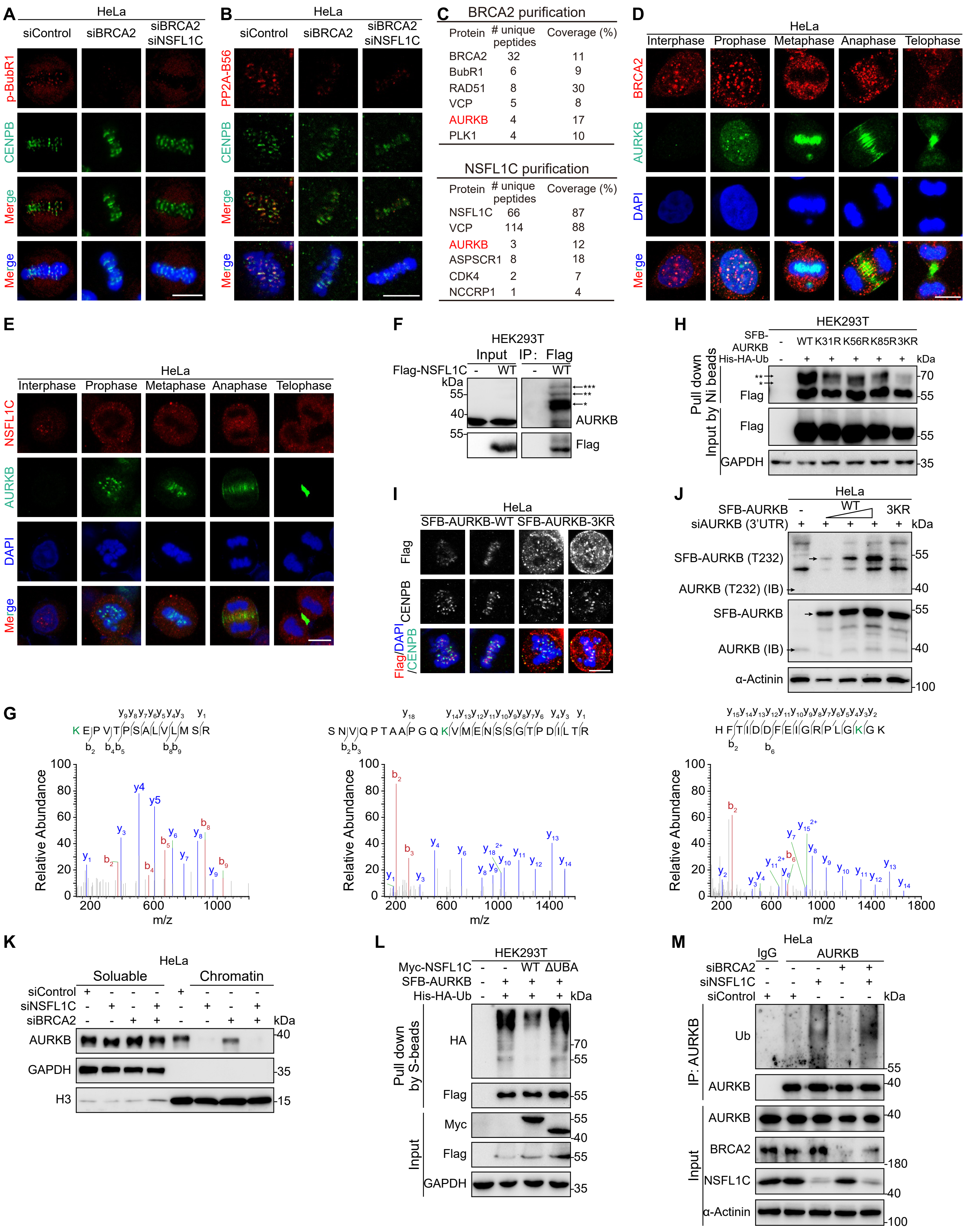

Supplemental Figure 4 (related to Figure 4). NSFL1C prevents premature dissociation of AURKB from the centromeres by decreasing polyubiquitination of AURKB. (**A–B**) Loss of NSFL1C did not restore p-BubR1 or PP2A-B56 foci formation in metaphase of BRCA2-deficient HeLa cells. Scale bar, 10 µm. (**C**) TAP-MS identified BRCA2-, and NSFL1C-associated proteins in HEK293T cells. See Supplementary Table 3 for details. (**D–E**) Representative images of BRCA2 and NSFL1C foci formation during mitosis in HeLa cells. Scale bar, 10 µm. (**F**) NSFL1C interacted with ubiquitinated AURKB. *, monoubiquitination; **, diubiquitination; ***, triubiquitination. Note that AURKB was ubiquitinated, displaying an 8-kDa shift. (**G**) TAP-MS identified the ubiquitylation site (lysine 31, 56 and 85) on the human AURKB protein in HEK293T cells. See Supplementary Table 3 for details. (**H**) Mutations in K31, K56 or K85 impaired AURKB ubiquitination levels. *, monoubiquitination; **, diubiquitination. (**I**) Representative images of Figure 4**F**. Scale bar, 10 µm. (**J**) AURKB-3KR mutant had a lower level of AURKB-T232ph, which is the direct marker of AURKB activity. HeLa cells were treated with siRNA targeting the 3'UTR region of *AURKB*, synchronized by nocodazole treatment and collected for immunoblotting detection. The black arrow points to endogenous AURKB (or T232) or exogenous AURKB (or T232) with SFB tags. (**K**) Depletion of NSFL1C decreased AURKB chromatin loading. HeLa cells were treated with the indicated siRNA, synchronized by nocodazole treatment and collected for immunoblotting detection. (**L**) Increasing the expression level of NSFL1C-WT reduced AURKB polyubiquitination, but the UBA domain deletion mutant of NSFL1C had no effect. (**M**) NSFL1C regulated ubiquitination of AURKB independent of BRCA2 loss. HeLa cells were treated with the indicated siRNA, synchronized by nocodazole treatment and collected for immunoblotting detection.

Supplemental Figure 5

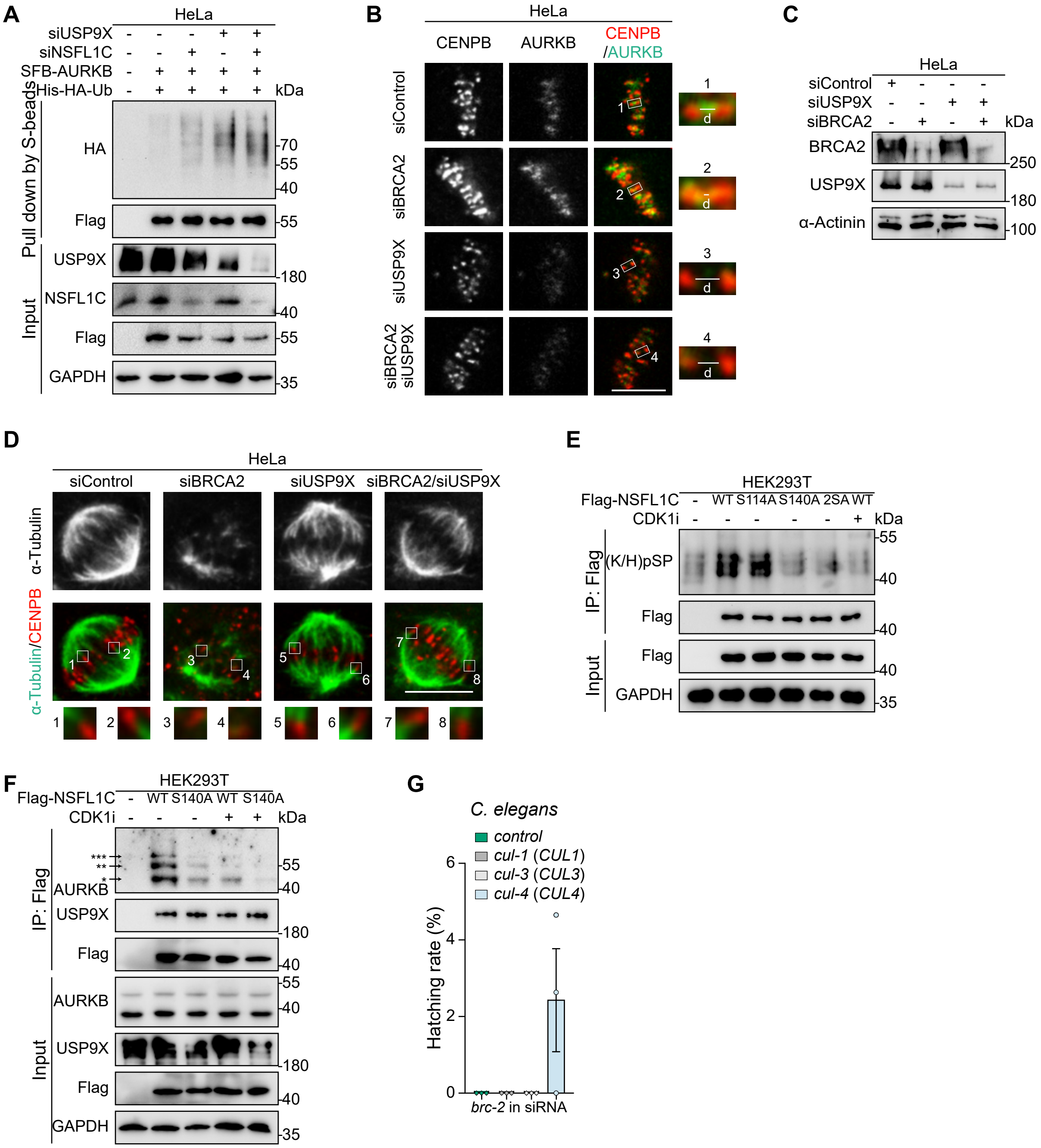

Supplemental Figure 5 (related to Figure 5). NSFL1C promotes USP9X mediated deubiquitination of AURKB to stabilize centromeric AURKB. (**A**) NSFL1C and USP9X regulated the ubiquitination of AURKB through the same pathway. HeLa cells were treated with the indicated siRNA, transfected with indicated plasmid, synchronized by nocodazole treatment and collected for immunoblotting detection. (**B**) Representative images of Figure 5**E**. AURKB, centromeric intermediate protein, as markers for locating centromeres; CENPB, as markers for centromeres. Insets show one enlargement of the outlined regions, and d represents the distance between centromeres. Scale bar, 10 μm. (**C**) Immunoblotting showing BRCA2 and NSFL1C depletion in HeLa cells. (**D**) Representative images of cold-stable microtubules of Figure 5**F**. HeLa cells were co-stained with α-tubulin and CENPB, as markers for centromeres. Insets show one enlargement of the outlined regions. Scale bar, 10 μm. (**E**) CDK1 phosphorylated the S140 site of the NSFL1C protein. HEK293T cells were transfected with indicated plasmid, synchronized by CDK1i (RO3306, 9 μM) treatment and collected for immunoblotting detection. (**F**) The interaction between NSFL1C and AURKB depended on CDK1. HEK293T cells were transfected with indicated plasmid, synchronized by CDK1i (RO3306, 9 μM) treatment and collected for immunoblotting detection. *, monoubiquitination; **, diubiquitination; ***, triubiquitination. (**G**) Hatching rate assay upon treatment with endogenous siRNA directed against *cul-1*/*cul-3*/*cul-4* in *C. elegans brc-2* mutants (n = 3). Data indicate the mean ± SEM.

Supplemental Figure 6

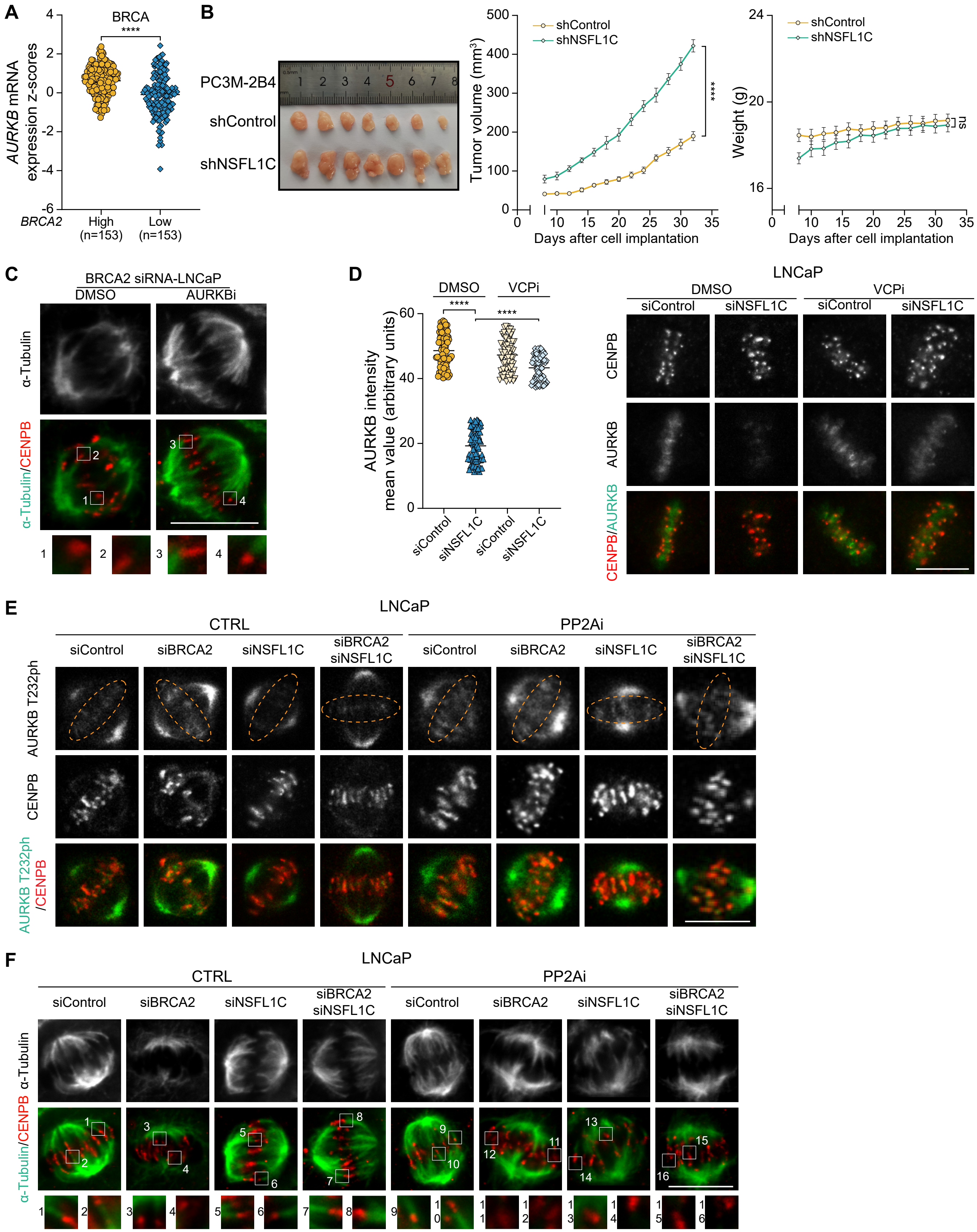

Supplemental Figure 6 (related to Figure 6). Inhibition of PP2A could reactive the SAC in BRCA2-deficient cells. (**A**) mRNA levels of *AURKB* were assessed in samples from high-*BRCA2* expression and low-*BRCA2* expression breast invasive ductal carcinoma (BRCA) patients in the TCGA dataset (from cBioPortal). Patients were separated into high-*BRCA2*/*AURKB* or low-*BRCA2*/*AURKB* on the basis of the 40th percentile of *BRCA2*/*AURKB* mRNA expression z-scores. (**B**) Defects in NSFL1C enhanced BRCA2-deficient tumors growth. PC3M-2B4 cells transfected with virus expressing the indicated shRNA were used in a xenograft tumor assay (n = 13/shControl 12/shNSFL1C). (**C**) Representative images of cold stable microtubules of Figure 6**E**. LNCaP cells were co-stained with α-tubulin and CENPB, as markers for centromeres. Insets show one enlargement of the outlined regions. Scale bar, 10 μm. (**D**) VCPi (NMS-873, 10 μM) rescued the accumulation of AURKB on the equatorial plate in *NSFL1C* knockdown LNCaP cells (n = 3). Scale bar, 10 μm. (**E**) Representative images of AURKB-T232ph intensity of Figure 6**F**, which is the direct marker of AURKB activity. Scale bar, 10 μm. (**F**) Representative images of cold-stable microtubules of Figure 6**G**. LNCaP cells were co-stained with α-tubulin and CENPB, as markers for centromeres. Insets show one enlargement of the outlined regions. Scale bar, 10 μm. Data indicate the mean ± SEM. Unpaired two-tailed Student's t test was used in **A**. Two-way ANOVA was used in **B** and **D**.

Supplemental Figure 7

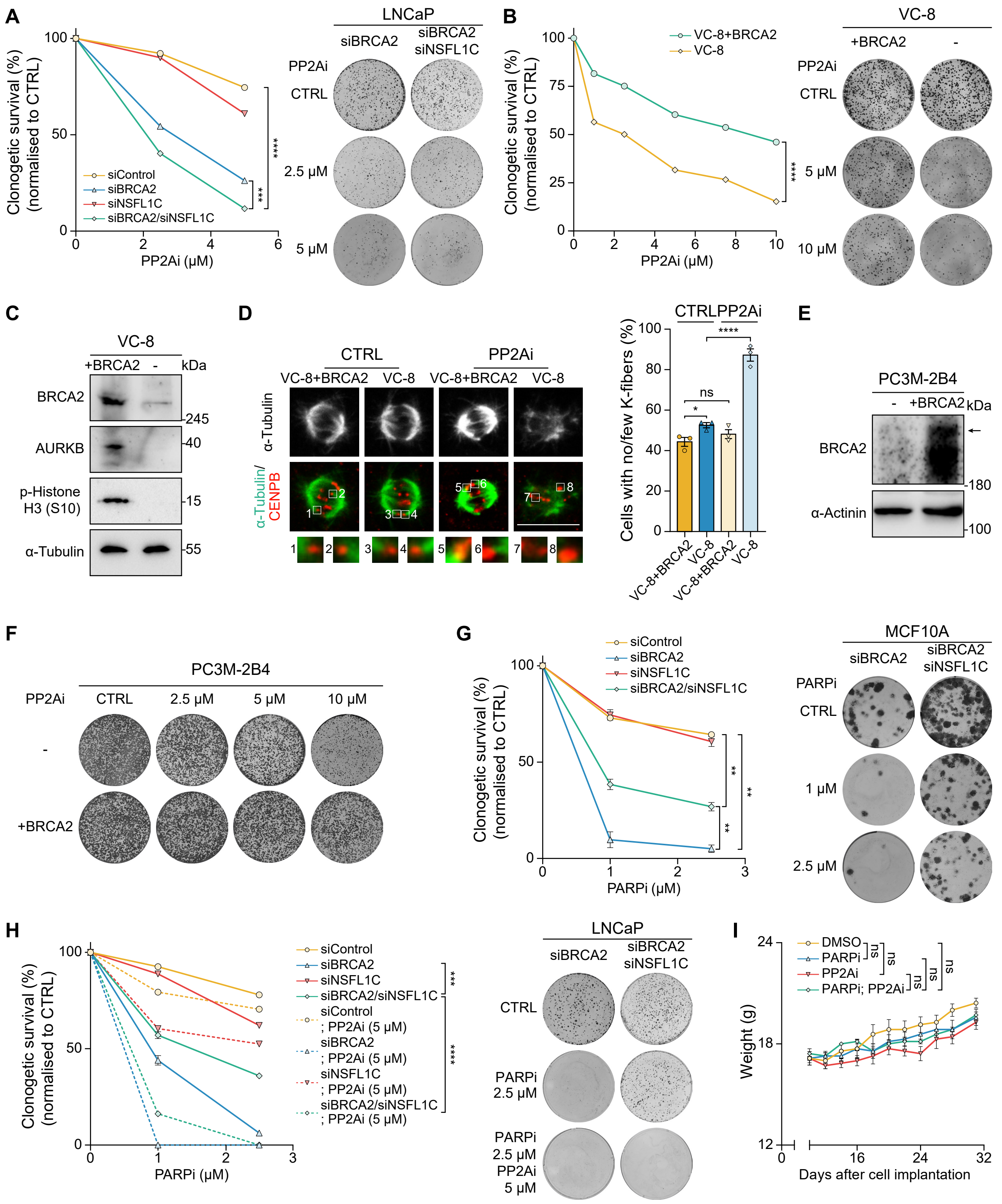

Supplemental Figure 7 (related to Figure 7). PP2A is an attractive synthetic lethal therapeutic target for *BRCA2*-mutated cancers. (A) LNCaP cells expressing the indicated siRNA were treated with the control or indicated dose of PP2Ai (LB100) (24 h), and cell survival rates were counted by calculating the colony numbers (n = 3). (**B**) VC-8/VC-8 + BRCA2 cells were treated with the control or indicated dose of PP2Ai (LB100) (24 h), and cell survival rates were counted by calculating the colony numbers (n = 3). (**C**) BRCA2 defective cells enhanced bypass SAC. VC-8 and VC-8 + BRCA2 cells were synchronized by nocodazole treatment and cells were collected for H3Ser10p immunoblotting detection, which is used as a marker for mitosis. (**D**) Cold-stable microtubules in VC-8 and VC-8 + BRCA2 cells treated with the indicated siRNA and PP2Ai (LB100). The frequency of K-fiber defects (n = 3) (right), and the representative images (left). Cells were co-stained with α-tubulin and CENPB, as markers for centromeres. Insets show one enlargement of the outlined regions. Scale bar, 10 μm. (**E**) Immunoblotting showing exogenous overexpression of BRCA2 in PC3M-2B4 cells. The black arrow points to the position of the BRCA2 protein. (**F**) Representative images of Figure 7**D**. (**G**) MCF10A cells expressing the indicated siRNA were treated with the control or indicated dose of PARPi (olaparib) (24 h), and cell survival rates were counted by calculating the colony numbers (n = 3). (**H**) PP2Ai and PARPi have synergistic therapeutic effects. LNCaP cells expressing the indicated siRNA were treated with the control, indicated dose of PP2Ai (LB100) or indicated dose of PARPi (olaparib) (24 h), and cell survival rates were counted by calculating the colony numbers (n = 3). (**I**) The weights of the nude mice used in Figure 7**G** (n = 7 or 8/group). Data indicate the mean ± SEM. Unpaired two-tailed Student's t test was used in **D** (CTRL). Two-way ANOVA was used in **A**, **B**, **D**, **G**, **H** and **I**.

Supplementary materials and methods

C. elegans RNA extraction. Up to 100 μl of worms were resuspended in 1 ml TRIzol (Thermo Fisher, 15596026), rapidly frozen in liquid nitrogen, and thawed at 37 °C. Repeated freeze–thaw cycles were carried out until the nematode split. After the addition of 200 μl chloroform, the samples were shaken by hand for 15 s, followed by an incubation of 2–15 min at room temperature and 12,000 g centrifugation at 4 °C for 15 min. The upper aqueous phase was taken and 0.5 ml isopropanol was added to the tube, followed by an incubation of 5–10 min at room temperature and 12,000 g centrifugation at 4 °C for 10 min. The upper aqueous phase was removed and an equal volume of 75% ethanol was added to clean the RNA precipitation. The samples were eluted in RNA-free water.

Antibodies. Antibodies used for this study were: Flag (Sigma, F3165), c-Myc (Santa Cruz, sc-9E10), RAD51 (gift from Dr. Jun Huang, Zhejiang University) (68), Cyclin A (Santa Cruz, sc-271682), BRCA2 (Millipore, OP95, IF), BRCA2 (Abcam, ab123491, IB), BRCA2 (Abclonal, A2435, IB), NSFL1C (Santa Cruz, sc-365215, IF), NSFL1C (Abclonal, A6677, IB), NSFL1C (Abcepta, AP22235a, IHC/IP), AURKB (BD, 611082), AURKB (Abclonal, A19539), AURKB (Abcam, ab2254, IHC), AURKB T232 (CST, 2914S), HSP70 (Abclonal, A12948), Histone H3 (Biodragon, B1055), GAPDH (Sungen, KM9002), KU80 (CST, 2180S), α-Tubulin (Sigma, T6199), α-Tubulin (Proteintech, 11224-1-AP), phospho-Histone H2A.X (Ser139) (CST, 9718S), CENPB (Santa Cruz, sc-376283), CENPB (Abcam, ab25734), Ub (Abclonal, A19686), BubR1 (Abcam, ab209998), BubR1 T680 (Abcam, ab200061), PP2A B56 (BD, 610615), USP9X (Abclonal, A9782), ACTN1 (Abclonal, A1160), (K/H) pSP (CST, 9477S), BrdU (BD, 347580), BrdU (Abcam, ab6326), H3Ser10p (CST, 9701S), HA (Abclonal, AE008), CDC20 (Santa Cruz, sc-5296), CDC20 (Proteintech, 10252-1-AP), UBXN2B (Origene, TA502489S), Biotin (Jackson, 200-002-211).

Inhibitors. Inhibitors used for this study were: Camptothecin (HARVEYBIO, 5 or 10 nM as indicated), Hydroxyurea (HARVEYBIO, 4 or 8 mM as indicated), Cisplatin (TargetMol 1 or 1.5 μM as indicated), Thymidine (TargetMol, 2 mM), Nocodazole (TargetMol, 40–100 ng/ml as indicated), CDK1i (RO3306, TargetMol, 9 μM), AURKBi (Barasertib, Selleck, 10 nM), MG132 (Selleck, 10 μM), VCPi (NMS-873, Selleck, 10 μM), PP2Ai (LB100, Selleck, 1, 2.5, 5 7.5 or 10 μM as indicated), PARPi (olaparib, Selleck, 1 or 2.5 μM as indicated), Monastrol (TargetMol, 100 μM).

Vector construction and generation of human stable cell lines and knockouts. cDNAs of *NSFL1C* and *AURKB* were subcloned into pDONR201 (Invitrogen) as entry clones and subsequently transferred to gateway-compatible destination vectors for expression of N-tagged fusion proteins. All deletion and point mutants were generated by PCR and verified by sequencing. MBP-BRCA2 (gift from Dr. Weibin Wang, Peking University). cDNAs of *Ceubxn-2*/*Ceair-2*/*Cebub-1*/*Cecul-1*/*Cecul-3*/*Cecul-4*/*Cemak-2* were cleaved and cloned into the pL4440 vector using T4 ligase (TransGen Biotech, FL101-02). HEK293T/HeLa cells stably expressing SFB-tagged constructs were generated by transfection of pDEST-puro containing the SFB-tagged construct. Selection began at 48 h using 2 μg/ml puromycin. Knockouts were generated in HCT116, U2OS and HeLa cells by transfecting pSpCas9 (BB)-2A-puro (p459). Selection began at 48 h using 2 μg/ml puromycin. All *BRCA2*/*NSFL1C* knockout cells were confirmed by TA cloning sequencing (TransGen Biotech, CT101-01) and immunoblotting. The sequences are shown in Supplementary Table 1.

siRNA and plasmid transfection. siRNAs were obtained from GenePharma and transfected using Lipofectamine RNAiMAX (Thermo Fisher, 13778150) according to the manufacturer's protocol. Plasmid transfections were carried out using polyethylenemine (Polysciences, 23966)

according to the manufacturer's protocol. For siRNA and DNA cotransfections, plasmids were transfected 6 h after siRNA treatment. The sequences are shown in Supplementary Table 1.

Lentivirus generation and transduction. The lentivirus was produced by transfecting HEK293T cells with helper plasmids pMD2G (Addgene, 12259) and psPAX2 (Addgene, 12260) using polyethylenemine at a mass ratio of 3:4:4 for plasmid DNA: pMD2G: psPAX2. The medium was replaced 6–8 h post transfection, and the viral supernatant was collected several times within 24–72 h of transfection. The supernatant was passed through a 0.22 μm PVDF filter before use (Millipore, GSWP02500). The lentivirus was added to target cell lines with 8 μg/ml polybrene (Yeasen, 40804ES76), and the medium was changed 15 h post infection. Antibiotics (2 μg/ml puromycin, 750 μg/ml G418 and 10 μg/ml blasticidin) were added 15 h post infection when selection was needed. For all biological replicates, independent lentivirus infection and antibiotic selection were performed each time.

Flow cytometry analysis. Flow cytometry was performed using a Calibur2 instrument (BD Biosciences). For the cell cycle assay, HeLa cells were trypsinized, centrifuged, fixed with 70% ice cold ethanol and stored at 4 °C. Twelve hours later, the fixed cells were permeabilized with 0.2% Triton X-100 and stained with propidium iodine (PI) staining solution (Sigma, P4170). To quantify the percentage of M phase cells, the fixed cells were permeabilized with 0.3% Triton X-100, incubated with anti-H3Ser10p primary antibody (1:100, CST, 9701S) for 30 min and then stained with secondary antibody conjugated with Alexa Fluor 488 (1:500, Thermo Fisher, A11008) for 30 min before PI staining.

Immunoprecipitation and immunoblotting. For analysis of the protein interactions in mitosis, nocodazole (100 ng/ml) treated HeLa or HEK293T cells were lysed. NETN buffer (20 mM Tris-HCl pH 8.0, 100 mM NaCl, 1 mM EDTA, and 0.5% Nonidet P-40) was used to lyse cells at 4 °C for 20 min with shaking. After the removal of cell debris by centrifugation (14,000 rpm for 10 min

at 4 °C), the soluble fractions were collected and incubated with S-protein agarose (Merck-Millipore, 69704) for 4 h at 4 °C. The S-protein agarose beads were washed three times with NETN buffer and boiled with 2× SDS loading buffer at 100 °C for 8 min.

For immunoprecipitation of endogenous CDC20, BRCA2, AURKB, Flag or USP9X, nocodazole treated cells were lysed in NETN buffer. After centrifugation, the lysate was incubated with CDC20 (Santa Cruz, sc-5296), BRCA2 (Abcam, ab123491), AURKB (Abcam, ab2254), Flag (Sigma, F3165) or USP9X (Abclonal, A9782) antibody or control IgG (Thermo Fisher, I5006 or 10400C) 3 h at 4 °C, and the lysate was incubated for an additional 1 h at 4 °C after adding 20 μl Protein A agarose (Merck-Millipore, 16-125). The Protein A agarose beads were washed three times with NETN buffer and boiled with 2× SDS loading buffer at 100 °C for 8 min. The samples were then subjected to SDS–PAGE and immunoblotting with specific antibodies.

Soluble fractions and chromatin fraction extraction. The soluble fractions were acquired by using NETN buffer. After centrifugation (14,000 rpm for 10 min at 4 °C), the remaining fractions were washed with PBS at least 3 times and lysed in cold EBC2 buffer (50 mM Tris-HCl pH 7.5, 300 mM NaCl, 5 mM $CaCl_2$, and 10 U microcal nuclease). After sonication for approximately 20 s and centrifugation at 14,000 rpm for 15 min at 4 °C, the supernatants were transferred to a fresh tube as chromatin fractions.

DNA fiber assay. HeLa cells depleted of BRCA2 and/or NSFL1C were labeled with 40 μM CldU (Sigma, C6891) for 20 min, washed with PBS and exposed to 100 μM IdU (Sigma, I7125) for 20 min. After exposure to IdU, the cells were washed again in warm PBS and treated with 4 mM HU 5 h before collection. Cells were then lysed, and DNA fibers were stretched onto glass slides, as described. The slides were fixed in a solution (methanol: acetic acid = 3:1) at -20 °C for 1 h, and fixed it at 4 °C for 20 min with 75% ethanol. The fibers were denatured with 2.5 M HCl for 1 h, washed with PBS and blocked with 2% BSA in PBS (0.1% Tween-20) for 30 min. The

newly replicated CldU and IdU tracts were revealed with anti-BrdU antibodies BU1/75 (1:500, Abcam, ab6326), and B44 (1:50, BD, 347580) recognizing CldU and IdU respectively. The fibers were then washed with PBS (0.1% Tween-20) and incubated with fluorescently tagged goat anti-rat Alexa Fluor 488 (1:500, Thermo Fisher, A11006) and goat anti-mouse Alexa Fluor 594 (1:500, Thermo Fisher, A11005) secondary antibodies for 1 h at RT. Images were taken at 63× magnification, and statistical analysis was performed using GraphPad Prism.

Immunofluorescence (IF). Nonsynchronized cells cultured on glass coverslips were fixed with 4% polyformaldehyde in PBS for 15 min at RT, washed three times with PBS (0.1% Tween-20) and permeabilized with 0.25% Triton X-100 in PBS for 10 min at RT. The cells were then washed, blocked for 30–60 min in PBS (2% BSA) at RT and incubated with rabbit anti-RAD51 (1:500, Dr. Jun Huang, Zhejiang University) and mouse anti-Cyclin A (1:500, Santa Cruz, sc-271682) for 1 h at RT. The cells were then washed with PBS (0.1% Tween-20) and incubated with fluorescently tagged goat anti-rabbit Alexa Fluor 488 (1:500, Thermo Fisher, A11008) and goat anti-mouse Alexa Fluor 594 (1:500, Thermo Fisher, A11005) secondary antibodies for 1 h at RT. The cells were washed in PBS (0.1% Tween-20) and stained with DAPI (0.4 μg/ml, Sigma-Aldrich) for 5 min at RT. After three washes in PBS (0.1% Tween-20), the coverslips were mounted on microscope slides.

For staining of γH2AX foci, BRCA2 and NSFL1C in mitosis, synchronized cells were treated with 40 ng/ml nocodazole and released at 0, 20, 40 and 60 min. The cells were treated for 4 h followed by fixation as described above for mitotic localization of γH2AX, BRCA2 and NSFL1C. γH2AX was detected by rabbit anti-phospho-Histone H2A.X (Ser139) (1:500, CST, 9718S), BRCA2 was detected by mouse anti-BRCA2 (1:50, Millipore, OP95) and NSFL1C was detected by mouse anti-NSFL1C (1:50, Santa Cruz, sc-365215). α-Tubulin was detected by mouse anti-α-Tubulin (1:1000, Sigma, T6199) or rabbit anti-α-Tubulin (1:200, Proteintech, 11224-1-AP).

Detection of mitotic EdU foci previously reported (21). Hela cells were synchronized at the G1/S transition with 2 mM thymidine for 18 hours, washed four times with PBS and released in fresh medium for 9 hours. Cells were labelled with 20 mM EdU (Bidepharm, BD103898) during the final hour, and processed. Cells were treated with as described above, and then performed a Click reaction (10 ml: 8.8 ml PBS, 50 µl 5 mM biotin-PEG3-azide (Sigma, 762024), 1 ml 100 mM sodium ascorbate (Sigma, A4034) and 200 µl 100 mM $CuSO_4 \cdot 5H_2O$ (BBI, A600063)), blocked, mouse anti-Biotin (1:500, Jackson, 200-002-211), goat anti-mouse Alexa Fluor 594 and stained with DAPI.

Kinetochore localization and cold-stable kinetochore–microtubules: synchronized cells treated with 100 µM Monastrol (TargetMol, T4048) 4 h and washed twice in PBS before 1–2 h incubation in media containing 10 µM MG132 (Selleck, S2619). After synchronization, the cells were fixed with 100% methanol for 3 min at -20 °C, rinsed once in PBS before fixation on ice with precooled 4% polyformaldehyde for 10 min. AURKB was detected by rabbit anti-AURKB (1:50, Abclonal, A19539) or mouse anti-AURKB (1:200, BD, 611082), HEC1 was detected by mouse anti-HEC1 (1:300, Abcam, ab3613), p-BubR1 was detected by rabbit anti-p-BubR1 (T680) (1:100, Abcam, ab200061), PP2A-B56 was detected by mouse anti-PP2A-B56 (1:250, BD, 610615), p-AURKB was detected by rabbit anti-p-AURKB (T232) (1:50, CST, 2914S) and Flag was detected by mouse anti-Flag (1:5000, Sigma, F3165). CENPB was detected by mouse anti-CENPB (1:50, Santa Cruz, sc-376283) or rabbit anti-CENPB (1:500, Abcam, ab25734) antibodies. To detect cold-stable microtubules, cells were synchronized using the above protocol, followed by 15 min of cold treatment on ice before fixation.

Live-cell imaging. HeLa cells expressing mRFP-Tubulin and GFP-H2B were seeded onto petri dishes with a 35 mm glass base (NEST, YA0570), grew in DMEM supplemented with 10% FBS, synchronized by double thymidine block, released and cultured for 4 h in normal growth

medium before filming was started. Fluorescence time-lapse images were taken every 5 min for 12 h at 37 °C in 5% CO_2 by using a laser-scanning confocal microscope (LSM780, Zeiss), at 40× magnification. Adobe Photoshop CC 2018 and Adobe Premiere Pro CC 2018 were used for image processing.

Immunohistochemistry (IHC). Aurora kinase B (AURKB) or NSFL1C immunohistochemical studies were performed on 4 μm thick formalin-fixed paraffin-embedded (FFPE) prostate carcinoma tissue sections obtained from 32 patients. Of the 32 patients, 10 had hereditary prostate carcinoma with a deleterious germline mutation of *BRCA2*, and 22 had sporadic prostate carcinoma without *BRCA2* mutation. *BRCA2* mutations were confirmed by next-generation sequencing (NGS) targeting a panel of homologous recombination genes. In the process of immunohistochemistry, antigen retrieval was performed using an EDTA-based solution at pH 9.0 for 20 min. An AURKB monoclonal antibody (1:200, Abcam, ab2254) or NSFL1C polyclonal antibody (1:200, Abcepta, AP22235a) was used. We calculated the extent and intensity of AURKB/NSFL1C-positive staining using Image-Pro Plus 6.0 software. The final expression degree of AURKB/NSFL1C was determined by the log-changed value of integral optical density (IOD). Two-tailed Student's t tests were used to compare the difference in the expression level of AURKB/NSFL1C between the *BRCA2* mutation group and the *BRCA2* wild type group.

Supplementary Table 1. List of siRNA, sgRNA and shRNA used in this study, related to materials and methods.

**siRNA**	**sgRNA**
siControl:<br>5’-CGUACGCGGAAUACUUCGA-3’	sgControl:<br>5’-GCGTGACATATATCGCGGGT-3’
siNSFL1C:<br>5’-GAGAGACCAGUAAACCGAGACCA-3’	sgNSFL1C:<br>5’-CGCTTCTTTCTCGAGTCGGC-3’
siUSP9X:<br>5’-ACUUCCUACCGAAUGCAGA-3’	sgBRCA2:<br>5’-ACGTTCGGGTTGTCCCTGGA-3’
siBRCA2:<br>5’-GAAGAAUGCAGGUUUAAUA-3’	
siUBXN2B:<br>5’-CUCCAGAAGAGGAGGAUAA-3’	**shRNA**
siBRCA1:<br>5’-CUAGAAAUCUGUUGCUAUG-3’	shControl:<br>5’-CGTACGCGGAATACTTCGA-3’
siATM:<br>5’-AACAUACUACUCAAAGACA-3’	shBRCA2:<br>5’-CAGGACACAATTACAACTAAA-3’
siAURKB (3'UTR):<br>5’-ACCUCCUCCUUUGUUUAAU-3’	shNSFL1C:<br>5’-GGGATGAAGACATTGTGACCA-3’

Supplementary Table 2. *C. elegans* second-generation genome exon sequencing differences between 95C19 and N2, *brc-2*/hT2.

info	chr	ref	alt	genotype	quality	MQ	ref-depth	alt-depth	Subtract *brc-2* -1	Subtract *brc-2* -2	Subtract N2
epg-2	chrI	A	T	hom	47	46	0	39	2302545	2302545	#N/A
ubr-4	chrI	G	A	hom	39	45	0	36	4627204	4627204	#N/A
W10G11.19	chrII	G	A	hom	35	46	0	34	#N/A	3551208	3551208
Y8A9A.2	chrII	A	T	hom	217	23	1	168	3796757	3796757	3796757
B0034.5	chrII	T	C	hom	37	39	0	35	5974584	5974584	5974584
mrps-18.C	chrII	A	C	hom	42	31	0	39	6736534	6736534	6736534
mrps-18.C	chrII	C	A	hom	41	31	0	37	6736536	6736536	6736536
mrps-18.C	chrII	A	C	hom	41	31	0	40	6736537	6736537	6736537
B0495.5	chrII	A	C	hom	48	30	0	46	7697470	7697470	7697470
B0495.5	chrII	C	T	hom	48	30	0	46	7697472	7697472	7697472
tbc-14	chrII	G	T	hom	35	46	0	31	13440675	13440675	13440675
smi-1	chrIII	T	G	hom	34	46	0	32	#N/A	1879552	1879552
fbxa-73	chrIII	A	T	hom	29	46	0	25	2253925	2253925	2253925
frm-8	chrIII	C	T	hom	41	46	0	37	#N/A	#N/A	#N/A
Y53G8AR.8	chrIII	T	C	hom	43	45	0	40	#N/A	#N/A	#N/A
hecw-1	chrIII	T	C	hom	34	46	0	33	3387045	3387045	3387045
acy-3	chrIII	C	T	hom	48	46	0	44	#N/A	#N/A	#N/A
pelo-1	chrIII	C	T	hom	32	46	0	29	4202510	4202510	#N/A
zmp-1	chrIII	C	T	hom	36	45	0	34	#N/A	#N/A	#N/A
nono-1	chrIII	A	G	hom	54	30	1	50	5953952	5953952	5953952
nono-1	chrIII	T	A	hom	53	30	0	51	#N/A	5953953	5953953
nono-1	chrIII	G	T	hom	53	30	0	51	5953957	5953957	5953957
dig-1	chrIII	G	A	hom	44	46	0	39	6787416	6787416	#N/A
R01H2.4	chrIII	C	T	hom	41	45	0	36	#N/A	#N/A	#N/A
F54H12.5	chrIII	C	T	hom	33	46	0	33	7970661	7970661	#N/A
prp-8	chrIII	C	G	hom	35	46	0	35	8165282	8165282	#N/A
B0303.7	chrIII	G	C	hom	26	39	0	23	8693701	8693701	8693701
ZK507.1	chrIII	C	T	hom	14	38	0	13	9101256	9101256	9101256
mut-7	chrIII	C	T	hom	37	46	0	36	#N/A	#N/A	#N/A
C07A9.10	chrIII	C	G	hom	30	46	0	29	9685894	9685894	#N/A
col-93	chrIII	C	A	hom	25	46	0	22	10985548	10985548	#N/A
tra-1	chrIII	C	T	hom	28	44	0	25	#N/A	#N/A	#N/A
ttm-1	chrIII	T	G	hom	28	46	0	28	12953329	12953329	12953329
mak-2	chrIV	C	G	hom	37	46	0	32	#N/A	#N/A	#N/A
Y41D4B.11	chrIV	C	T	hom	31	46	0	27	1607211	#N/A	#N/A
ubxn-2	chrIV	C	T	hom	37	46	0	34	#N/A	#N/A	#N/A
cla-1	chrIV	A	C	hom	58	24	2	45	7591643	7591643	7591643
cla-1	chrIV	G	A	hom	12	23	0	11	#N/A	7592922	7592922
cla-1	chrIV	A	C	hom	27	31	1	25	7596309	7596309	7596309
cla-1	chrIV	A	T	hom	7	26	0	7	7596427	7596427	7596427
cla-1	chrIV	T	A	hom	78	21	14	53	7597783	#N/A	7597783
cla-1	chrIV	A	G	hom	6	23	0	4	#N/A	7599695	7599695
col-125	chrIV	A	C	hom	40	44	0	39	11226271	11226271	11226271
cyp-31A2	chrIV	A	G	hom	40	46	0	37	#N/A	11405088	11405088
Y58A7A.4	chrV	G	A	hom	5	29	0	5	#N/A	5084757	5084757
Y58A7A.4	chrV	C	A	hom	18	36	0	15	5084806	5084806	5084806
ttn-1	chrV	T	G	hom	7	25	0	6	6177100	6177100	6177100
ttn-1	chrV	A	T	hom	138	29	13	113	6178476	6178476	6178476
oac-29	chrV	G	A	hom	37	45	0	34	#N/A	8633529	8633529
B0507.7	chrV	G	T	hom	35	39	0	33	8757776	8757776	8757776
B0507.7	chrV	T	G	hom	35	40	0	35	8757777	8757777	8757777
nhr-44	chrV	C	G	hom	35	46	0	33	#N/A	8963428	8963428
str-45	chrV	C	T	hom	36	45	0	30	#N/A	15159894	15159894
Y39B6A.25	chrV	G	A	hom	38	46	0	35	#N/A	19029747	19029747
gap-1	chrX	T	C	hom	35	46	0	33	#N/A	#N/A	2192531
C12D12.1	chrX	C	T	hom	33	46	0	32	#N/A	#N/A	3502807
F35A5.1	chrX	G	A	hom	8	33	0	6	3812044	3812044	3812044
zig-2	chrX	C	A	hom	34	46	0	30	12341341	12341341	12341341

Supplementary Video 1. (separate file):

Time-lapse imaging of cell division treated with siControl, related to Figure 2**C**.

Supplementary Video 2. (separate file):

Time-lapse imaging of cell division treated with siBRCA2, related to Figure 2**C**.

Supplementary Video 3. (separate file):

Time-lapse imaging of cell division treated with siNSFL1C, related to Figure 2**C**.

Supplementary Video 4. (separate file):

Time-lapse imaging of cell division treated with siBRCA2/siNSFL1C, related to Figure 2**C**.

Supplementary Table 3. (separate file):

Mass Spectrometric analysis results of BRCA2, NSFL1C and AURKB, related to Supplementary Figure 4, **C** and **G**, and Figure 5**A**.
